# Long-term effects of repeated multitarget high-definition transcranial direct current stimulation combined with cognitive training on response inhibition gains

**DOI:** 10.3389/fnins.2023.1107116

**Published:** 2023-03-09

**Authors:** Zhihua Guo, Rui Qiu, Huake Qiu, Hongliang Lu, Xia Zhu

**Affiliations:** Department of Military Medical Psychology, Air Force Medical University, Xi’an, China

**Keywords:** transcranial direct current stimulation, stop-signal task, cognitive training, response inhibition, neuroplasticity, right inferior frontal gyrus, pre-supplementary motor area

## Abstract

**Background:**

Few studies have investigated the effects of repeated sessions of transcranial direct current stimulation (tDCS) combined with concurrent cognitive training on improving response inhibition, and the findings have been heterogeneous in the limited research. This study investigated the long-lasting and transfer effects of 10 consecutive sessions of multitarget anodal HD-tDCS combined with concurrent cognitive training on improving response inhibition compared with multitarget stimulation or training alone.

**Methods:**

Ninety-four healthy university students aged 18–25 were randomly assigned to undergo different interventions, including real stimulation combined with stop-signal task (SST) training, real stimulation, sham stimulation combined with SST training, and sham stimulation. Each intervention lasted 20 min daily for 10 consecutive days, and the stimulation protocol targeted right inferior frontal gyrus (rIFG) and pre-supplementary motor area (pre-SMA) simultaneously with a total current intensity of 2.5 mA. Performance on SST and possible transfer effects to Stroop task, attention network test, and N-back task were measured before and 1 day and 1 month after completing the intervention course.

**Results:**

The main findings showed that the combined protocol and the stimulation alone significantly reduced stop-signal reaction time (SSRT) in the post-intervention and follow-up tests compared to the pre-intervention test. However, training alone only decreased SSRT in the post-test. The sham control exhibited no changes. Subgroup analysis revealed that the combined protocol and the stimulation alone induced a decrease in the SSRT of the low-performance subgroup at the post-test and follow-up test compared with the pre-test. However, only the combined protocol, but not the stimulation alone, improved the SSRT of the high-performance subgroup. The transfer effects were absent.

**Conclusion:**

This study provides supportive evidence for the synergistic effect of the combined protocol, indicating its superiority over the single intervention method. In addition, the long-term after-effects can persist for up to at least 1 month. Our findings also provide insights into the clinical application and strategy for treating response inhibition deficits.

## 1. Introduction

Response inhibition comprises the ability to withhold irrelevant or context-inappropriate responses following changes in the environment so that one can make flexible and goal-directed behavioral responses, which is one of the core components of executive function (Verbruggen and Logan, 2009; Diamond, 2013). It is an essential factor for self-adaptation and self-regulation of the dynamics of actions (Aron, 2007; Sandrini et al., 2020). Response inhibition is closely associated with many other cognitive abilities, such as impulse control, working memory (WM), and cognitive inhibition (Dalley and Robbins, 2017; Zhao et al., 2018; Xu et al., 2020; Weidler et al., 2022). It is commonly impaired in many psychiatric disorders, such as substance use disorder, psychopathy, attention deficit hyperactivity disorder (ADHD), and schizophrenia (Hughes et al., 2012; van Rooij et al., 2015; Kohl et al., 2019; Gillespie et al., 2022).

Due to its great importance, the neural substrates and the approach to enhancing response inhibition have recently received increasing attention. Accumulating evidence has identified a frontal-basal ganglia network engaged in response inhibition, including the right inferior frontal gyrus (rIFG), the pre-supplementary motor area (pre-SMA), and the basal ganglia (Aron and Poldrack, 2006; Duann et al., 2009; Aron et al., 2014; Hannah and Aron, 2021). Transcranial direct current stimulation (tDCS) is a promising and widely used neuromodulatory technique for regulating cortical activity and neuroplasticity and enhancing cognitive function (Nitsche and Paulus, 2001; Pisoni et al., 2018). It is a suitable tool to infer the causality for the links between brain function and corresponding behavioral changes (Filmer et al., 2014; Gbadeyan et al., 2016; Yavari et al., 2018). tDCS is safe, non-invasive, tolerable, and easy-to-operate (Bikson et al., 2016) and has been found to effectively enhance response inhibition *via* anodal stimulation targeting rIFG or pre-SMA (Hsu et al., 2011; Jacobson et al., 2011; Ditye et al., 2012; Kwon and Kwon, 2013b,a; Stramaccia et al., 2015; Sandrini et al., 2020; Fujiyama et al., 2021).

New forms of tDCS emerge as research into the effect of tDCS on enhancing response inhibition progresses. High-definition tDCS (HD-tDCS) is an optimized form of conventional pad-tDCS with high spatial precision and produces more prominent behavioral and neurophysiological effects (Kuo et al., 2013; Sehatpour et al., 2021). Multitarget stimulation refers to simultaneous stimulation with the same polarity on multiple functionally related brain cortices, which can modulate the cortical activity more efficiently and enhance tDCS effects more prominently than conventional single-target stimulation (Hill et al., 2018; Gregoret et al., 2021; Guo et al., 2022a). Given behavioral and neuroimaging evidence, a previous study has shown that multitarget high-definition stimulation of rIFG and pre-SMA is more effective in improving response inhibition compared with the commonly used single-target stimulation on rIFG or pre-SMA alone (Guo et al., 2022a).

Importantly, repeated sessions of tDCS can increase efficacy through cumulative effects, yield long-lasting after-effects and stable changes in brain function, and are tolerated and safe (Nitsche et al., 2008; Cohen Kadosh et al., 2010; Paneri et al., 2016; Turski et al., 2017; Di Rosa et al., 2019; Song et al., 2019). Since cognitive training and tDCS both modulate neuroplasticity, combining tDCS and related cognitive training that involves the same or similar neural network may generate a synergistic and additional effect (Elmasry et al., 2015; Val-Laillet et al., 2015; Allenby et al., 2018; Berryhill and Martin, 2018; Wilkinson et al., 2019; Schneider et al., 2021). This combined approach can affect the trained tasks and be generalized to other untrained cognitive functions (transfer effect), including near and far transfer effects (Filmer et al., 2017a; Berryhill and Martin, 2018; Brem et al., 2018; Forcano et al., 2018; Smits et al., 2021).

However, limited studies focused on whether repeated tDCS combined with concurrent behavioral task training further extends response inhibition performance relative to a single intervention method, and the findings are heterogeneous among these few studies. Some studies have shown that this combination can induce greater response inhibition enhancement or better clinical outcomes (improved abstinence rate of alcohol), with the effects lasting 1 or 2 weeks (Dousset et al., 2021; Dubuson et al., 2021). However, according to some findings, this combination cannot produce additional benefits for response inhibition performance at post-intervention or follow-up sessions (Smits et al., 2021; Westwood et al., 2021; Zhou and Xuan, 2022). Additionally, the near and far transfer effects generated by this combined approach have scarcely been explored and warrant further studies. For instance, a previous study using tDCS together with stop-signal task (SST) training found that non-trained task (implicit association task) showed no evidence of intervention effects (Smits et al., 2021). To date, no researchers have investigated the effect of repeated daily multitarget tDCS (a new stimulation montage) combined with concomitant cognitive training on extending performance improvements of response inhibition. In addition, its long-term after-effects and transfer effects should be examined.

To fill the research gap, we designed this study to investigate the effects of 10 consecutive sessions of multitarget anodal HD-tDCS targeting rIFG and pre-SMA combined with concurrent cognitive training on improving response inhibition compared with 10 repeated sessions of multitarget stimulation or training alone, including long-lasting effects and transfer effects. Based on available research, we hypothesized that (1) the combined approach would extend and enhance performance improvements of response inhibition compared to multitarget stimulation or cognitive training alone, and the improvement effects would persist to follow-up session (i.e., long-term after-effect), (2) multitarget stimulation or cognitive training alone would induce response inhibition improvements compared to sham tDCS, and (3) the transfer effects would be absent. To the best of our knowledge, this study is the first to examine the effects of repeated daily multitarget anodal HD-tDCS combined with concurrent cognitive training on response inhibition, providing a preliminary insight into strategies to enhance response inhibition ability for both psychiatric and non-psychiatric populations.

## 2. Materials and methods

### 2.1. Participants

Ninety-four healthy university students were included in this study. Prior to inclusion, the participants were screened to ensure they were ≥18 years of age and unfamiliar with tDCS-related research. They reported no neuropsychiatric disorders or use of psychotropic medication. All the participants (*n* = 94, mean age = 20.88 ± 1.77 years, range = 18–25 years, 41 males) had a normal or corrected-to-normal vision, no contraindications to tDCS (e.g., metal implants in the head, open wounds in the scalp, a family or personal history of epilepsy), and were right-handed as assessed with the Edinburgh Handedness Inventory (Oldfield, 1971). The participants were also evaluated in hyperactivity/impulsivity and inattention using the Adult ADHD Self-report Scale (ASRS), and only those with scores of <17 in both subscales were included because individuals with a score of ≥17 on either subscale were likely to have ADHD (Kessler et al., 2005; Yeh et al., 2008). The participants were randomly assigned to four groups: (1) real stimulation combined with SST training, *n* = 24 (stimulation + training group); (2) real stimulation, *n* = 21 (stimulation group); (3) sham stimulation combined with SST training, *n* = 24 (sham + training group); and (4) sham stimulation, *n* = 25 (sham control). Each group underwent intervention separately, without knowing each other. We used G*power 3.1.9.6 to compute a prior sample size with a medium effect size of 0.25, two-tailed α of 0.05, and power (1-β) of 0.80, and a sample of 52 participants was planned (13 per group) (Cohen, 1992; Faul et al., 2007). Written informed consent was obtained from all the participants after the experimental procedure was explained to them. They were free to withdraw from the study at any stage. All the experimental protocols were reviewed and approved by the Tangdu Hospital Ethics Committee, Air Force Medical University, and were performed under the Declaration of Helsinki. After finishing the experiment, the participants received monetary compensation for their time.

### 2.2. Design and procedure

**FIGURE 1:**
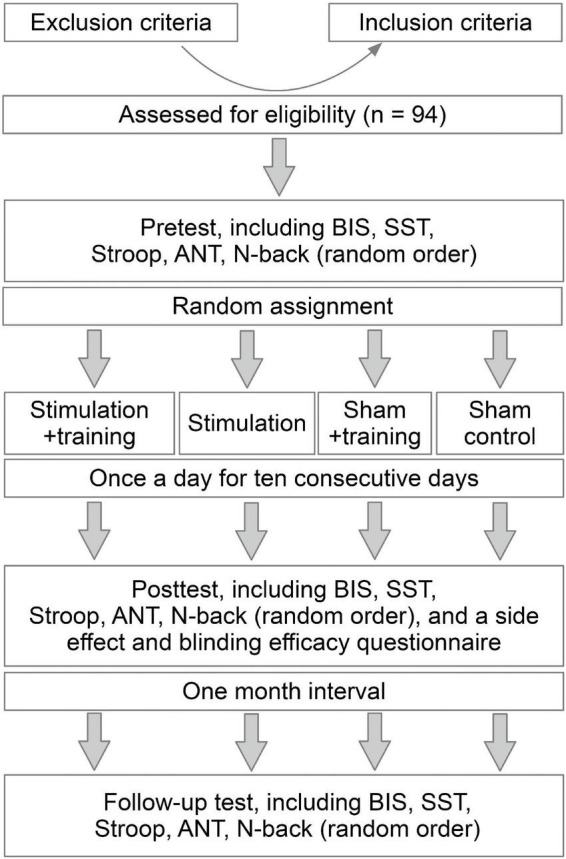
Experimental procedure. The study followed a single-blind, randomized, parallel-group, and sham-controlled design. The order of SST, Stroop task, ANT, and N-back task were randomized.

The current study had a single-blind, randomized, parallel-group, and sham-controlled design. The participants were blind to the intervention conditions and study hypotheses. Before undertaking the experiment, the participants were asked to complete a brief questionnaire to collect their demographic information, the ASRS scores, and assess their eligibility for tDCS. There were 13 sessions in this study: pre-intervention test, 10 intervention sessions, post-intervention test, and a follow-up test after a month. After the pre-test, the participants were randomly assigned to four intervention conditions. Each participant received 10 sessions of corresponding intervention for 20 min per day on 10 consecutive days. The training did not start until a stable holding current was obtained to avoid the confounding effect of current fluctuations (Zhou and Xuan, 2022). Side effects and blinding efficacy were evaluated *via* interviews with the participants after finishing the intervention sessions. All the participants completed the measurements before the intervention (pre-intervention test), the day after the end of the intervention (post-intervention test), and 1 month after intervention (follow-up test). The test contents were identical every time (Figure 1), including the Barratt Impulsiveness Scale-Version 11 (BIS-11), SST, color-word Stroop task, N-back task, and attention network test (ANT). The BIS-11 lasted for about 5 min; the test SST, Stroop task, and N-back task each lasted for about 10 min; the ANT lasted for about 16 min. In addition to SST, which assessed response inhibition, other tasks examined the potential transfer effects (near transfer: Stroop task; far transfer: N-back task and ANT). Before each measurement, BIS-11 was used to assess changes in self-reported impulsivity. The tasks were computerized and run on E-prime 3.0 software (Psychology Software Tools, Inc., Sharpsburg, PA, USA). The behavioral tasks were administered in a randomized order (Martin et al., 2013; Dubuson et al., 2021). Before beginning each task, the participants were instructed on how to perform the task; then, a standardized written instruction appeared on the screen.

### 2.3. High-definition transcranial direct current stimulation

**FIGURE 2:**
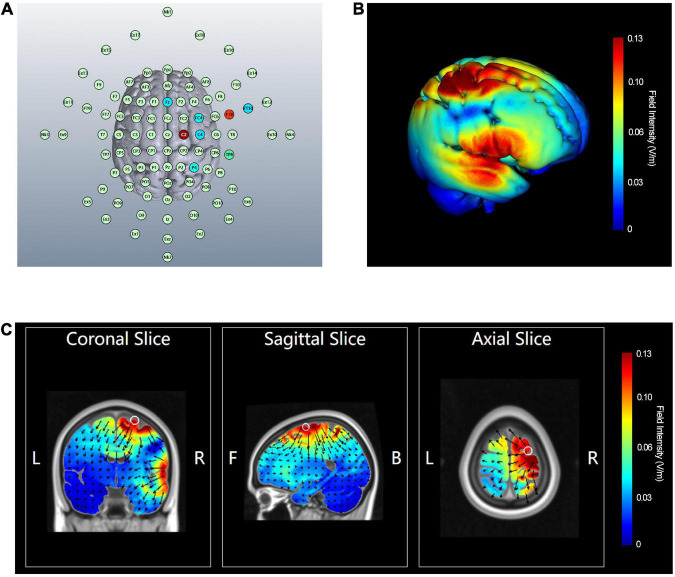
Electrode configuration and computational neurostimulation modeling of multitarget HD-tDCS. **(A)** Electrodes configuration. **(B)** A 3D view of the simulated electric field. **(C)** The section view of simulated electric field and current direction. The color bar represents the field intensity. The arrow points in the direction of the current flow, and the length indicates the current flow intensity. L, left; R, right; F, front; B, back.

Multitarget HD-tDCS was delivered using an M × N-9 HD-tES Stimulator (Soterix Medical, Inc., New York, NY, USA), following the procedures for HD-tDCS usage specified in a previous study protocol (Villamar et al., 2013). The stimulation procedure in this study used multitarget HD-tDCS on rIFG and pre-SMA from our previous study (Guo et al., 2022a). The electrodes were localized according to the international 10-10 EEG system (Jurcak et al., 2007). Anodes were placed at C2 (1.48 mA) and FT8 (1.02 mA) (a total current intensity 2.5 mA), with return cathodes at Fz (−0.51 mA), C4 (−0.52 mA), P4 (−0.36 mA), FT10 (−0.53 mA), TP8 (−0.17 mA), and FC4 (−0.41 mA) (Figure 2A). The electric field and current flow were simulated (Figures 2B, C and Supplementary Figures 1–5) using HD-explore and HD-Targets software (Soterix Medical, Inc., New York, NY, USA). This simulation method has been widely used in prior studies and proved effective (Shen et al., 2016; Stephens and Berryhill, 2016; Reinhart and Nguyen, 2019). Participants in the sham stimulation condition underwent the same procedure as the real stimulation condition. The panel of the instrument was not visible to the participants. The current intensity of each electrode was smaller than 1.5 mA, which has been shown to be safe and reliable enough to improve cognitive performance (Villamar et al., 2013; Bikson et al., 2016; Hogeveen et al., 2016; Abellaneda-Perez et al., 2021; Zhou et al., 2021). Real stimulation was applied for 20 min with a ramp-up of 30 s at the beginning and a ramp-down of 30 s at the end. Sham stimulation consisted of a 30 s ramp-up and a 30 s ramp-down at the beginning and end, respectively, with no current during the intervening time, facilitating blinding by mimicking the sensations of real tDCS without actual neurophysiological changes (Di Rosa et al., 2019; Sharma et al., 2021). After stimulation sessions, the participants guessed which kind of stimulation they received (real or sham) and rated the confidence level based on a numeric analog scale ranging from 0 = *absolute guess* to 10 = *absolutely sure*. Additionally, participants completed a side-effect survey to report their dominant sensations (e.g., itching, tingling, burning, metallic taste, no special sensation) during the stimulation, and an 11-point scale was used to evaluate the intensity of sensations they felt, ranging from 0 = *no sensation* to 10 = *strongest sensation imaginable* (Hill et al., 2017).

### 2.4. Tasks and measures

#### 2.4.1. Barratt impulsiveness scale-version 11

Barratt impulsiveness scale-version 11 was employed to evaluate the impulsivity of the participants. It comprises 30 items and can be divided into three dimensions: attentional impulsivity, motor impulsivity, and non-planning impulsivity, with 10 items in each dimension (Patton et al., 1995; Bari and Robbins, 2013). In the current study, we used the revised Chinese version of BIS-11 (Li et al., 2011). It is reliable and has been widely used in previous studies (Ran et al., 2021; Guo et al., 2022b). Each item can be rated from 1 to 5 based on a five-point Likert scale. The dimensional score and total score range from 0 to 100 after being converted, with higher scores indicating higher levels of impulsivity (Li et al., 2011; Ran et al., 2021). The internal consistency of the BIS scale and its three subscales were good in our sample, with the Cronbach’s α ranging from 0.70 to 0.91 at an arbitrary test time point.

#### 2.4.2. Stop-signal task

**FIGURE 3:**
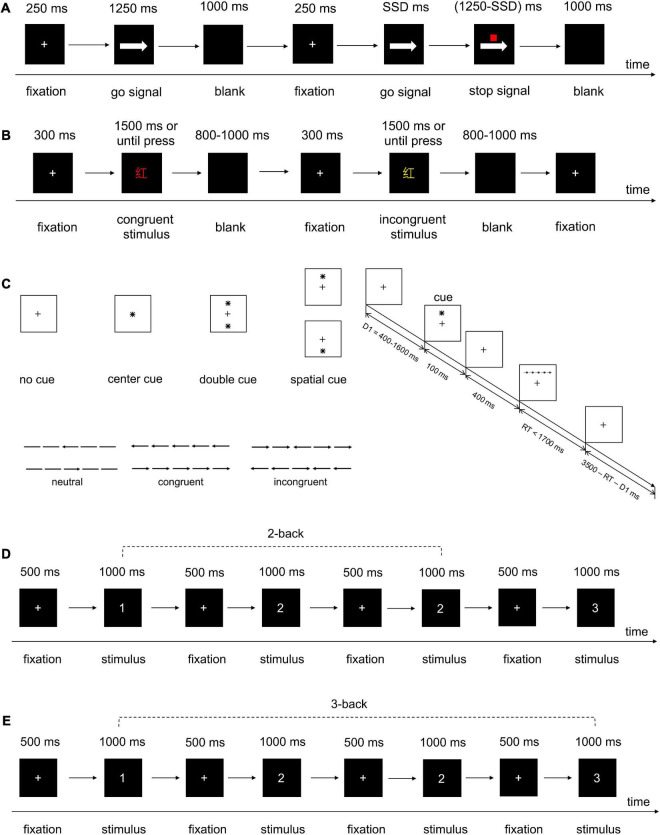
Detailed information about procedures of behavioral tasks. **(A)** SST. **(B)** Color-word Stroop task. **(C)** ANT. **(D)** 2-back task. **(E)** 3-back task.

We used SST to evaluate the response inhibition performance (Logan et al., 1984; Verbruggen and Logan, 2008; Verbruggen et al., 2019). The task settings were identical to our previous study (Guo et al., 2022a). In the pre-potent go trials (75% of total trials), the participants were instructed to discriminate the direction of the right arrow or left arrow go signal on the screen by pressing the corresponding key (F for the left arrow and J for the right arrow) on a standard keyboard as quickly and accurately as possible. However, in the stop trials (25% of total trials), a small red square (stop signal) was presented above the arrow after an interval (stop signal delay, SSD), indicating the need to withhold their initiated response. The SSD was dynamically adjusted stepwise (initial SSD = 250 ms, 50-ms step, range = 0–1250 ms) to ensure that each participant had an approximately 50% successful inhibition rate. Figure 3A presents the details of the task parameters. We estimated the primary outcome measure using the stop-signal reaction time (SSRT) determined by the integration method (Verbruggen et al., 2019), with shorter SSRT indicating superior response inhibition. SSRT was determined as follows: (1) calculating p(response| stop-signal), which means the probability of response to a stop signal; (2) ranking all RT of go trials from the minimum to the maximum with go omissions assigning the maximum RT (RT distribution); (3) calculating nth RT which corresponds to the p(response| stop-signal)-percentile of the RT distribution; and (4) using nth RT minus mean SSD to calculate SSRT. In addition to SSRT, other SST performance metrics, such as stop accuracy (the probability of inhibiting responses on stop stimulus) and goRT (mean RT on correct go trials), were also assessed.

The SST was not only the test task for all groups but also the training task for the two groups using SST training. The test SST included a practice block of 48 trials and a formal test block of 200 trials (25% stop-signal trials), while the training SST consisted of 48 practice trials and 400 formal trials (30-s rest when finishing 200 trials). The training SST finished within the stimulation duration to guarantee the identical training amount. All the trials were presented at random.

#### 2.4.3. Color-word Stroop task

The participants performed a classical color-word Stroop task at the pre-test, post-test, and follow-up test, which is a measure of cognitive inhibition (Lu et al., 2020a; Parris et al., 2021; Wu et al., 2021b; Zhou and Xuan, 2022). The Stroop task was used to explore the near-transfer effect of various interventions on cognitive inhibition. The task included a practice block of 15 trials and two test blocks of 45 trials each, with a 30-s rest between formal experimental blocks. The stimulus was chosen randomly from one of three Chinese characters (“红” for red, “绿” for green, and “黄 for yellow) printed in different colors of ink, either red, green, or yellow (Lu et al., 2020a). The practice block was presented with feedback, and the participants did not proceed to the formal test block until 80% accuracy was achieved. The formal test block had no feedback. Each trial began with a fixation cross (+) at the center of the screen for 300 ms, which was replaced by a Stroop stimulus. The participants were instructed to press “D” for red, “F” for yellow, and “J” for green on the keyboard, according to the color rather than the meaning of the Chinese character, as quickly and accurately as possible. The stimulus interface lasted up to 1500 ms or was terminated with a blank screen (800–1000 ms) immediately after a key-press response (Figure 3B). During the congruent trial, the word matched the color (e.g., “红” in red), while in the incongruent trial, the word conflicted with the ink color (e.g., “红” in yellow). In our task, 40% of trials were incongruent, and all the trials were presented randomly (Fu et al., 2019). We adopted the Stroop effect as the primary outcome. It was characterized by a longer reaction time in incongruent conditions compared with color-word congruent conditions and measured by the mean correct RT in incongruent trials, subtracting the mean correct RT in congruent trials. A lower Stroop effect indicated a higher inhibitory performance (Stroop, 1935; Fu et al., 2019; de Boer et al., 2021).

#### 2.4.4. Attention network test

Attentional network test (ANT) is a classic task to study attention ability, which simultaneously measures the efficiency of individual alerting, orienting, and executive control networks involved in attention (Fan et al., 2002; Goldin et al., 2014; Lu et al., 2020b). The ANT was used to measure the transfer effect on attentional function. In our study, the ANT featured identical visual and timing parameters to those previously described (Fan et al., 2002). The target was preceded with one of the four cues, namely no cue, center cue, double cue, and spatial cue, and was flanked on either side by two arrows pointing in the same direction (congruent condition), opposite direction (incongruent condition), or no direction (neutral condition). The participants were asked to identify the direction (left/right) of the targeted arrow in the upper or lower visual hemifield by pressing a corresponding key (“F” for the left arrow, “J” for the right arrow) as quickly and accurately as possible. A session included a 24-trial practice block and two test blocks of 96 trials each (Rinne et al., 2013). The participants did not enter the test block until 60% accuracy of the practice block was achieved. The trials were presented in a random order. There was a 30-s rest between two experimental blocks to avoid mental fatigue in the participants. Figure 3C presents more details. Outcome measures included the following: (1) conflict effect = RT (incongruent)–RT (congruent); (2) orienting effect = RT (central cue)–RT (spatial cue); and (3) alerting effect = RT (no cue)–RT (double cue) (Fan et al., 2002). The higher the orienting and alerting effects, the better the attentional processing; the lower the conflict effect, the better the ability to deal with interference.

#### 2.4.5. N-back task

To probe the far transfer effect on the WM, we used an N-back task that is widely used to measure WM performance (Owen et al., 2005; Alizadehgoradel et al., 2020; Kaminski et al., 2020). We used a 2-back combined with a 3-back task with two blocks of each kind of task, and the 2-back task was conducted before the 3-back task. A cue appeared before each task block to alert the participants whether the next block was a 2-back or 3-back block. A number stimulus ranging from 1 to 9 appeared on the screen every time, and the participants were instructed to press the “J” key when the targets were identical to the ones presented two numbers before in a 2-back task block or three numbers before in a 3-back task block; otherwise, they pressed “F” in the keyboard. There were 62 trials in a 2-back task block and 63 trials in a 3-back task block, and the participants could have a 30-s rest between blocks. The participants had to finish the practice block before the test block started. Figures 3D, E present the details of the time sequence of the trials. The mean RT of correct responses and response accuracy were assessed as a result, and shorter RT and higher accuracy rates indicated better WM performance (Alizadehgoradel et al., 2020; Nejati et al., 2020).

### 2.5. Data pre-processing

Concerning SST, five participants were excluded from further analyses because they showed (1) stop accuracy <0.25 or >0.75 or (2) SSRT <50 ms (Congdon et al., 2012). After exclusion, the sample for SST analysis consisted of 89 subjects (*n* = 23, 21, 22, 23 for groups 1 to 4, respectively). Five participants were excluded from the Stroop effect analysis due to RT exceeding ± 3 SD of the mean (Fu et al., 2019). After exclusion, the Stroop task analysis was based on *n* = 23, 21, 22, 23 for groups 1 to 4, respectively. As for the N-back task, four participants with accuracy or RT exceeding ± 3 SD of the mean were excluded, leaving 90 participants for further analyses (*n* = 23, 20, 23, 24 for groups 1 to 4, respectively). Concerning ANT, five participants were excluded due to RT deviating >3 SDs of the mean. The final sample for ANT analysis comprised 89 participants (*n* = 22, 21, 21, 25 for groups 1 to 4, respectively). Notably, the number of participants varied by measure because of data filtering of corresponding behavioral measures, which was common practice in previous studies (Biggs et al., 2015; Dagan et al., 2018).

### 2.6. Statistical analysis

We used the IBM SPSS statistical package version 26 to conduct data analyses. The normality in the distribution of data was evaluated using the Shapiro-Wilk test, and the homogeneity of variances was confirmed using Levene’s test. When necessary, the sphericity assumption was verified by Mauchly’s sphericity test, and Greenhouse-Geisser was applied when the sphericity assumption was not met. Categorical variables such as gender and blinding were represented as count or proportion and examined by the chi-squared test. Continuous variables such as accuracy and RT were presented as mean ± standard deviation (SD). One-way analysis of variance (ANOVA) was used to test baseline performance and continuous data that measured once such as demographic variables. If the outcome measures differed at baseline (i.e., pre-test), they were analyzed by creating contrasts (δ values) between the post-test or follow-up test and pre-test to eliminate the interference of baseline, thereby ensuring that any performance changes would be attributable to the intervention. In addition, one-way ANOVA with Bonferroni’s-corrected statistical threshold was used to test group differences of δ_post–pre_ or δ_follow–up–pre_.

Each behavioral task and its outcome measures and BIS-11 scores were tested using a series of 4 × 3 repeated-measures ANOVA (RM-ANOVA) with group (stimulation + training/combined condition, stimulation, sham + training, sham control) as between-subject factor and time (pre-test, post-test, follow-up test) as within-subject factor. *Post-hoc* tests were performed using Bonferroni’s-corrected pairwise comparisons. To further detect the effects of different intervention conditions on improving response inhibition, we conducted a subgroup analysis of SSRT. The participants in each group were separated into high-performance (HP) and low-performance (LP) subgroups based on baseline SSRT *via* a median-split method (Whelan et al., 2012; Schmicker et al., 2021). Subgroup analysis for each condition was performed using a 2 (subgroup: HP and LP) × 3 (time: pre-test, post-test, and follow-up test) RM-ANOVA. To explore possible relationships between SST and other behavioral tasks, we computed correlations of baseline outcome measures (excluding participants according to data filtering criteria of both tasks) using bivariate Pearson’s correlation analysis (two-tailed test). For exploring purposes, the statistical threshold of correlation analysis was not corrected. Concerning RM-ANOVAs, the significant interaction term was the focus of this study. The statistical significance level was set at α = 0.05. For ANOVAs, partial eta-squared (η_*p*_^2^) was calculated as measure of effect sizes.

## 3. Results

### 3.1. Demographics and baseline performance

As shown in Table 1, the four groups were matched. There were no significant differences in demographic and basic characteristics between the groups (*p*s > 0.05), including gender distribution, age, years in education, scores of hyperactivity/impulsivity and inattention subscales of ASRS, and sleep duration per night. In addition, one-way ANOVA for scores of BIS-11 and outcome measures of SST, Stroop task, N-back task, and ANT revealed no significant differences in the variables at baseline between the groups (*p*s > 0.05), except for 2-back accuracy, 3-back accuracy, and orienting effect (Table 1).

**TABLE 1:** Demographic data, scale scores, and behavioral tasks performance at baseline.

Variable	Stimulation + training	Stimulation	Sham + training	Sham control	F/χ^2^	*p*
*n*	24	21	24	25		
Gender (male/female)	10/14	10/11	10/14	11/14	0.213	0.976
Age (years)	20.83 (1.74)	20.71 (1.95)	20.88 (1.75)	21.08 (1.73)	0.170	0.917
Education (years)	15.42 (1.77)	15.24 (1.79)	15.46 (1.93)	15.64 (1.73)	0.192	0.902
ASRS-inattention	12.00 (2.83)	12.57 (2.01)	13.17 (2.53)	12.24 (2.51)	0.983	0.404
ASRS-hyperactivity/impulsivity	9.33 (2.88)	9.00 (2.92)	9.46 (2.86)	9.64 (3.16)	0.187	0.905
Sleep duration per night (hours)	7.00 (0.83)	6.81 (0.87)	6.75 (0.74)	6.64 (0.57)	0.968	0.412
**BIS-11**						
Non-planning impulsivity	28.65 (14.52)	30.83 (13.45)	31.88 (13.48)	28.20 (11.78)	0.416	0.742
Motor impulsivity	29.27 (9.22)	32.62 (8.27)	32.50 (8.20)	32.60 (10.29)	0.788	0.504
Attentional impulsivity	31.88 (9.00)	34.76 (9.74)	33.02 (6.84)	29.40 (8.14)	1.644	0.185
**SST**						
SSRT (ms)	274.73 (28.47)	272.06 (32.82)	277.56 (34.75)	274.03 (36.57)	0.101	0.959
Stop accuracy	0.51 (0.07)	0.51 (0.04)	0.53 (0.06)	0.50 (0.06)	1.337	0.268
GoRT (ms)	565.20 (202.08)	506.71 (152.52)	569.66 (216.79)	497.66 (221.20)	0.796	0.499
**Stroop task**						
Stroop effect (ms)	114.58 (47.34)	121.60 (67.99)	131.96 (72.02)	100.98 (57.96)	1.005	0.395
**ANT**						
Orienting effect (ms)	122.74 (27.01)	132.02 (24.90)	107.10 (30.62)	127.16 (32.99)	2.899	0.040
Conflict effect (ms)	51.34 (32.41)	56.25 (20.94)	39.24 (26.98)	50.42 (28.06)	1.438	0.237
Alerting effect (ms)	52.42 (32.14)	54.35 (25.96)	45.88 (25.19)	50.95 (27.54)	0.356	0.785
**N-back task**						
2-back accuracy	0.82 (0.07)	0.68 (0.22)	0.71 (0.12)	0.70 (0.18)	3.207	0.027
2-back RT (ms)	652.56 (72.61)	657.90 (96.93)	685.37 (65.40)	657.05 (81.27)	0.815	0.489
3-back accuracy	0.73 (0.11)	0.69 (0.12)	0.62 (0.10)	0.71 (0.15)	3.652	0.016
3-back RT (ms)	640.07 (74.14)	660.87 (57.69)	651.52 (97.77)	611.24 (111.20)	1.335	0.268

Values are counts or means (standard deviations). ASRS, adult ADHD self-report scale; BIS-11, Barratt impulsiveness scale-version 11; SST, stop-signal task; SSRT, stop-signal reaction time; GoRT, mean reaction time on correct go trials; ANT, attention network test. The gender distribution was tested by the χ^2^ test, and other variables were examined using one-way analysis of variance.

### 3.2. HD-tDCS safety, blinding efficacy, and electric field simulation

All the participants tolerated the stimulation well, and only mild side effects (i.e., tingling, burning, itching) were reported. Most of the participants reported tingling sensation, with 19 (79.2%), 15 (71.4%), 21 (87.5%), and 21 (84.0%) subjects in groups 1 to 4, respectively. Moreover, there was no significant difference in the ratings of the intensity of tingling sensations between the four intervention conditions [*F*_(3,72)_ = 1.704, *p* = 0.174, η^2^_*p*_ = 0.066]. There were 24 (100%), 19 (90.5%), 23 (95.8%), and 24 (96%) participants in groups 1 to 4, respectively, who believed that they underwent real stimulation. No significant differences were found between the groups in the number of participants reporting real or sham stimulation (χ^2^ = 2.385, *p* = 0.45). The confidence level scores were also non-significant when they were compared between the stimulation + training (8.21 ± 1.29), stimulation (7.57 ± 2.40), sham + training (7.38 ± 2.16), and sham control (8.24 ± 1.76) conditions [*F*_(3,90)_ = 1.245, *p* = 0.298, η^2^_*p*_ = 0.04]. The electric field modeling showed that the electric field distribution generated by multitarget HD-tDCS was focused around the anodes and the electric field and current flow produced was largely restricted within the ring of return electrodes (Figures 2B, C).

### 3.3. Stop-signal task

**FIGURE 4:**
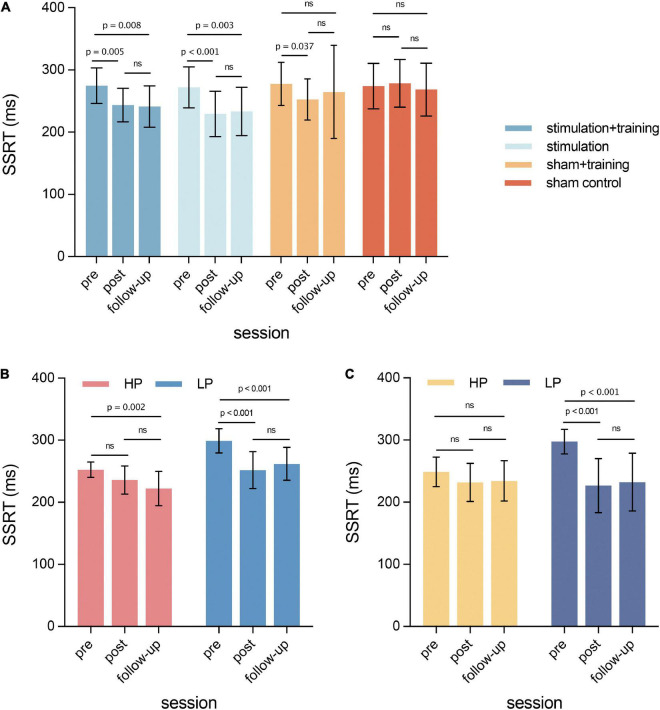
The effects of different intervention conditions in relation to the stop-signal task. **(A)** Significant interaction between group and time. **(B)** Subgroup analysis in the stimulation + training group. **(C)** Subgroup analysis in the stimulation group. HP, high performance; LP, low performance. All error bars represent standard deviation.

A significant group × time interaction effect on SSRT was observed [*F*_(6,170)_ = 2.161, *p* = 0.049, η^2^_*p*_ = 0.071] (Figure 4A). The main effects of time and group were also significant (*p*s < 0.05). *Post hoc* analysis with a Bonferroni’s-correction showed a significant decrease in SSRT both in the stimulation + training and stimulation alone groups form pre-intervention to post-intervention (*p* = 0.005 and *p* < 0.001, respectively) and from pre-intervention to follow-up test (*p* = 0.008 and *p* = 0.003, respectively). It also revealed a significant decrease in SSRT between pre-intervention and post-intervention in the sham + training group (*p* = 0.037) but not between pre-intervention vs. 1-month follow-up (*p* = 0.737). *Post hoc* analysis showed no significant changes in SSRT in the sham control group (*p*s > 0.999). There were no significant group × time interaction effects for the stop accuracy [*F*_(5.36,152.95)_ = 0.387, *p* = 0.869, η^2^_*p*_ = 0.013] and goRT [*F*_(5.42,153.69)_ = 0.776, *p* = 0.578, η^2^_*p*_ = 0.027], and the main effects were all non-significant (*p*s > 0.05).

Subgroup analysis showed a significant subgroup × time interaction for SSRT in both stimulation + training [*F*_(2,42)_ = 3.538, *p* = 0.038, η^2^_*p*_ = 0.144] and stimulation conditions [*F*_(2,38)_ = 5.105, *p* = 0.011, η^2^_*p*_ = 0.212]. The main effects of time and subgroup reached significance in the stimulation + training group (*p*s < 0.05), and the time main effect was significant in the stimulation group [*F*_(2,38)_ = 13.182, *p* < 0.001, η^2^_*p*_ = 0.41]. In the combined intervention (stimulation + training) condition, the Bonferroni’s-corrected *post hoc* analysis showed significantly decreased SSRT between pre-intervention and follow-up in the HP subgroup (*p* = 0.002), and between pre-test and post-test (*p* < 0.001) and between pre-test and follow-up test in the LP subgroup (*p* < 0.001) (Figure 4B). In the stimulation-alone condition, the SSRT significantly decreased in the post-test (*p* < 0.001) and follow-up test (*p* < 0.001) compared to the pre-test in the LP subgroup but not in the HP subgroup (Figure 4C). For the sham + training and sham control conditions, the interactions of subgroup × time were not significant (*p* = 0.214 and 0.098, respectively). The main effect of the subgroup was significant in the sham + training group [*F*_(1,20)_ = 4.568, *p* = 0.045, η^2^_p_ = 0.186]. There were no significant main effects in the sham control group (*p*s > 0.05).

### 3.4. Transfer tasks

In the Stroop task, the main effect of time was significant [*F*_(2,170)_ = 24.085, *p* < 0.001, η^2^_p_ = 0.221] due to decreased Stroop effect at the post-test (95.73 ± 5.44 ms) and follow-up test (73.36 ± 4.79 ms) compared to the pre-test (117.28 ± 6.53 ms). The interaction effect of group × time and the main effect of the group were not significant (*p*s > 0.05). In the ANT, one-way ANOVA showed that both the orienting effect δ values were not significant (*p*s > 0.05). The time effects for conflict [*F*_(1.82,155.04)_ = 11.705, *p* < 0.001, η^2^_p_ = 0.121] and alerting [*F*_(2,170)_ = 4.057, *p* = 0.019, η^2^_p_ = 0.046] effects were significant but not interaction terms or group effects (*p*s > 0.05). Concerning the N-back task, the baseline 2-back accuracy significantly differed between the combined intervention and stimulation conditions (Table 1), with the former exhibiting significantly higher accuracy than the latter (*p* = 0.047). One-way ANOVA showed that the δ_post–pre_ and δ_follow–up–pre_ for 2-back accuracy reached significance (*p* = 0.024 and 0.017, respectively, with corrected α = 0.025), but not 3-back accuracy (*p*s > 0.05). *Post hoc* analysis revealed that the combined intervention condition exhibited a smaller 2-back accuracy for δ_post–pre_ (*p* = 0.022) and δ_follow–up–pre_ (*p* = 0.013) compared to the stimulation condition. The main effects of time for 2-back RT and 3-back RT were significant (*p*s < 0.001) due to the reduction of RT at the post-test and follow-up test compared to the pre-test, but the interaction terms and the group effects were not significant (*p*s > 0.05).

### 3.5. Barratt impulsiveness scale-version 11

None of the group × time interactions and main effects of time and group for non-planning impulsivity, motor impulsivity, and attentional impulsivity reached significance (*p*s > 0.05).

### 3.6. Correlation analysis

Pearson’s correlation analysis showed that SSRT was significantly and negatively associated with 2-back (*r* = −0.259, *p* = 0.015) and 3-back (*r* = −0.239, *p* = 0.024) accuracy but was not correlated with the Stroop effect in the Stroop task or orienting, conflict, alerting effects in ANT (*p*s > 0.05).

## 4. Discussion

To the best of our knowledge, this randomized, parallel, and sham-controlled study is the first to examine whether repeated daily multitarget HD-tDCS applied to rIFG and pre-SMA, combined with concurrent SST response inhibition training, enhanced the response inhibition improvements. Consistent with the study hypotheses, our main findings showed that the combined protocol could generate a synergistic effect, compared to the single intervention condition, which also improved the response inhibition compared to the sham control. The decreased SSRT suggests improved response inhibition (Verbruggen and Logan, 2008; Verbruggen et al., 2019). According to the current results of SSRT, the combined protocol and the stimulation alone significantly improved response inhibition after the intervention, and the improvement persisted for up to at least 1 month. Given that the training alone only produced post-intervention effects, this condition was inferior to the combined condition and the stimulation alone in the long-term effects. However, the combined condition not only enhanced the LP subgroup performance but also improved the HP subgroup performance at the follow-up session compared to the stimulation-alone condition, which only enhanced the response inhibition of the LP subgroup. According to the compensation hypothesis (Shaw and Hosseini, 2021; Teixeira-Santos et al., 2022), the effects of cognitive enhancement techniques, such as tDCS and cognitive training, depend on baseline performance, and individuals with high baseline performance are difficult to be enhanced because they may already be near the peak level of cognitive ability. Therefore, there is less room for improvement. Conversely, individuals with low baseline performance have more room for improvement and are predisposed to enhancement. Many studies favor the compensation hypothesis (Krebs et al., 2021; Wu et al., 2021a,b; Assecondi et al., 2022). Despite the high baseline performance of the HP subgroup in this study, the combined protocol produced an improved effect at the follow-up session. Overall, the repeated daily HD-tDCS combined with SST training yielded the most significant effects and extended the improvement effects of stimulation or training alone.

The main finding is consistent with numerous previous studies that repeated tDCS accompanied by cognitive training could induce a synergistic effect after the intervention (Filmer et al., 2017b; Dousset et al., 2021; Dubuson et al., 2021; Schneider et al., 2021; Corrêa et al., 2022; Han et al., 2022; Lo et al., 2022; Szymkowicz et al., 2022). Importantly, the response inhibition improvement in this study persisted for up to 1 month following the intervention, consistent with previous studies in which repeated sessions of tDCS combined with concurrent cognitive training could produce after-effects that persisted from 1 week to 1 month (Dousset et al., 2021; Dubuson et al., 2021; Lee and Kim, 2021; Pisano et al., 2022). However, in the two studies involving response inhibition (Dousset et al., 2021; Dubuson et al., 2021), the after-effects lasted for 1 or 2 weeks, which differs from the 1-month after-effects in our study. This inconsistency may be attributed to the duration of intervention in previous studies that used four or five daily sessions of 20 min compared with 10 daily sessions of 20 min in this study. Most previous studies did not focus on the effects of combined condition on response inhibition, and among the few relevant studies, some findings rule out the synergistic effect of tDCS combined with response inhibition training (Smits et al., 2021; Westwood et al., 2021; Zhou and Xuan, 2022). However, our study provides evidence to support the higher efficacy of the combined protocol than commonly used single training or tDCS, providing further support for the limited literature on the efficacy of combined protocol in further improving response inhibition.

Previous studies have proposed that the best effects of tDCS are achieved when the stimulated neural network is already activated or pre-activated (e.g., *via* a behavioral task that involves the same brain region). Simultaneous activation of shared neural networks by both applied tDCS and performing relevant tasks can produce a synergistic effect. In addition, repeated tDCS and cognitive training may interactively facilitate the beneficial effect which occurs through specific neuroplastic changes such as the *N*-methyl-D-aspartate (NMDA)-dependent mechanism (Gilmore et al., 2018; Wilkinson et al., 2019; Breitling et al., 2020; Schneider et al., 2021; Westwood et al., 2021). The SST was widely used to study response inhibition and has been shown to engage the rIFG and pre-SMA (Aron and Poldrack, 2006; Duann et al., 2009; Watanabe et al., 2015; Hannah and Aron, 2021). Based on previous studies, we speculate that the neural mechanisms underlying the synergistic effect in our study may lie in the neural plasticity changes of the shared response inhibition cortices, including rIFG and pre-SMA, which were activated and shaped by the SST training and multitarget HD-tDCS. However, future studies are warranted, including the use of neuroimaging tools such as tDCS-compatible fMRI or magnetic resonance spectroscopy (MRS) to record simultaneous brain activity during the tDCS combined with SST training.

Additionally, we found that the repeated sessions of multitarget stimulation or SST training alone could improve response inhibition compared with the sham control condition, consistent with our hypothesis. The favorable effect of multitarget stimulation over sham control on response inhibition is in line with our previous study (Guo et al., 2022a). It is also similar to published studies indicating that multitarget stimulation exerted more significant effects on motor function than sham control (Dagan et al., 2018). Furthermore, this study explored the long-term effects of the multisession multitarget stimulation and found the improvement persisted for 1 month after intervention, similar to a previous study in which 10 repeated sessions of tDCS over dorsolateral prefrontal cortex (DLPFC) could improve task performance for 1 month after the intervention (Alizadehgoradel et al., 2020). This finding also showed that SST training alone improved response inhibition ability after the intervention. Not surprisingly, training is one of the crucial cognitive enhancers, and several studies have confirmed that SST training plays an important role in facilitating response inhibition (Berkman et al., 2014; Zhou and Xuan, 2022).

We found the good performance of SST was associated with high N-back accuracy at baseline, suggesting a correlation between response inhibition and WM in the mechanism. This is consistent with previous studies that at a behavioral level, response inhibition and WM are correlated (Alderson et al., 2010, 2017; Raiker et al., 2012), and at a functional level, response inhibition and WM both activate the rIFG (McNab et al., 2008). The scores of BIS-11 subscales measuring trait impulsivity showed no changes in this study, which is consistent with a previous study that revealed no variations of BIS-11 under the influence of time and intervention (training combined with either real or sham stimulation) (Gilmore et al., 2018). According to previous studies, personality traits increase in stability during puberty and remain relatively stable after that (Hayes et al., 2017). Therefore, the absence of an intervention effect is probably because the trait impulsivity assessed *via* BIS-11 remained relatively stable in our sample that comprised adults aged 18 years and older.

Although the 2-back accuracy δ values of the stimulation condition were higher than those of the combined protocol, this was attributed to the baseline difference between the two conditions. Since the 2-back accuracy of the combined protocol was significantly higher than the stimulation condition, it had less room for improvement (Shaw and Hosseini, 2021; Teixeira-Santos et al., 2022). Therefore, the difference was unrelated to the interventions. Overall, the transfer effects on the Stroop task, ANT, and N-back task, which measure cognitive inhibition, attentional function, and WM, respectively, showed no group differences attributable to the intervention. A previous study showed that seven daily sessions of SST training positively impacted the Stroop task performance, while the anodal stimulation on pre-SMA combined with SST training did not (Zhou and Xuan, 2022). This is partly consistent with our findings, but some discrepancy exists in that the SST training had no transfer effects in our study. This discrepancy might have arisen from the variations in the number of formal SST training trials; the SST training comprised 400 trials per session in our study, whereas the SST training consisted of 720 trials per session in the previous study. Furthermore, the total number of trials was less in our study (4000 vs. 5,040 trials). Concerning the transfer effect on attention and WM, previous studies have revealed that 10 online (i.e., tDCS concurrent with the task) sessions of tDCS + dual N-back training could produce a transfer effect to an untrained test of attention and WM at follow-up (Martin et al., 2013), or five sessions of multiple-task cognitive training with tDCS could lead to a near-transfer effect of attention gains (Boroda et al., 2020). However, no studies on online tDCS combined with response inhibition training have explored transfer effects on ANT or N-back. Therefore, they cannot be directly compared with our study. The transfer effect should be further considered and investigated.

In this study, to stimulate pre-SMA, we placed central anode at C2. A circuit was formed between the anode and cathodes, which led to current density and electric field existing between the electrodes—between the anode at C2 and the cathodes at Fz and FC4. The detailed simulation (Supplementary Figures 1–5) showed that the electric field extended through the anterior portion of Area 6 (Area 6a and 6ma) to the transition of Area 6 and Area 8 (Area i6-8 and s6-8). It cannot be excluded that parts of the motor area were stimulated as well, but fortunately this brain cortex has not been shown to be involved in the response inhibition process, which did not impact the interpretation of the findings in this study. Furthermore, there may be some confusions arising from the anode placement of pre-SMA because some previous studies placed the central anode at Fz to stimulate pre-SMA (Berglund-Barraza et al., 2020; Chiang et al., 2021). This is because there may be some ambiguity in what people are calling “pre-SMA.” We see that some places call Area 8 pre-SMA and others call the anterior Area 6 pre-SMA. Here we adopted the latter definition.

The current study has important theoretical and clinical implications. Regarding the theoretical implications, our findings support the synergistic effect of combining tDCS and concurrent cognitive training, indicating better improvement effects than the single intervention method. Moreover, we provided evidence that the combined protocol can be effectively applied in the field of response inhibition enhancement, with the long-lasting after-effects persisting for at least 1 month. Regarding the clinical implication, this study may provide insights into the treatment strategy for the clinical populations with inhibition-deficit-related mental diseases, who need to enhance response inhibition.

Despite these important implications, this study has some limitations. First, this study did not use neuroimaging method; therefore, we cannot infer the neural plasticity changes caused by the intervention. In the future, we plan to study brain functional and structural changes induced by this combined protocol. Second, the long-term after-effects were not investigated thoroughly. We only conducted a 1-month follow-up test, and further long-term effects were unknown, which should be dealt with in future studies. Third, this study focused on only young, healthy adults; therefore, it is not known how generalizable our findings are to other populations, such as the clinical sample, and the applicability of our results to other populations requires replication in other samples. Fourth, the study used a single-blinded design due to experimental constraints, possibly weakening the power of this study. Future studies should use more rigorous experimental designs to minimize potential bias, such as the Rosenthal effect. Fifth, the focality of multitarget anodal HD-tDCS in this study has to be improved. The electric field simulation result showed that the maximal electric field strength achieved underneath the anodes C2 and FT8, which we intended to stimulate pre-SMA and rIFG. However, the anode at C2 may also stimulated right motor cortex. Hence, in this study, the electric field produced by the stimulation protocol covered pre-SMA but the precision and focality were not enough, indicating the multitarget stimulation protocol needs to be improved to increase the focality of stimulation. Finally, due to the inter-individual variations of the cortical anatomy and reactivity to stimulation, the individual MRI data should be collected to improve the spatial localization accuracy and the individualized multitarget stimulation protocol for optimal effectiveness is highlighted, and this personalized application might be developed in the future.

## 5. Conclusion

The present study is the first to use multitarget stimulation combined with concurrent SST training to explore the enhanced improvement effect of response inhibition of this protocol compared to stimulation or training alone. We found that 10 daily sessions of combined interventions and the stimulation alone improved response inhibition, and the effects persisted for 1 month. The training alone only caused improved performance after the intervention. Furthermore, the combined protocol could modulate the performance of the individuals with high baseline response inhibition, which was not seen in the stimulation-alone condition. Notwithstanding the absence of transfer effects, it is too early to conclude that there is no transfer effect, and further studies are warranted. Thus, this study provides supportive evidence for the synergistic effect of the combined protocol. In addition, the long-term after-effect can persist for at least 1 month. Our findings also provide insights into the clinical application and strategy for treating response inhibition deficits.

## Data availability statement

The raw data supporting the conclusions of this article will be made available by the authors, without undue reservation.

## Ethics statement

The studies involving human participants were reviewed and approved by the Tangdu Hospital Ethics Committee, Air Force Medical University. The patients/participants provided their written informed consent to participate in this study.

## Author contributions

ZG and XZ: concept and design. ZG, RQ, HQ, and HL: acquisition, analysis, and interpretation of data. ZG: drafting of the manuscript. XZ: obtained funding. All authors contributed to the article and approved the submitted version.

## References

[B1] Abellaneda-Perez K. Vaque-Alcazar L. Perellon-Alfonso R. Sole-Padulles C. Bargallo N. Salvador R. (2021). Multifocal transcranial direct current stimulation modulates resting-state functional connectivity in older adults depending on the induced current density. *Front. Aging Neurosci.* 13:725013. 10.3389/fnagi.2021.725013 34899266 PMC8662695

[B2] Alderson R. Patros C. Tarle S. Hudec K. Kasper L. Lea S. (2017). Working memory and behavioral inhibition in boys with ADHD: an experimental examination of competing models. *Child Neuropsychol.* 23 255–272. 10.1080/09297049.2015.1105207 26563880

[B3] Alderson R. M. Rapport M. D. Hudec K. L. Sarver D. E. Kofler M. J. (2010). Competing core processes in attention-deficit/hyperactivity disorder (ADHD): do working memory deficiencies underlie behavioral inhibition deficits? *J. Abnorm. Child Psychol.* 38 497–507. 10.1007/s10802-010-9387-0 20140491

[B4] Alizadehgoradel J. Nejati V. Movahed F. S. Imani S. Taherifard M. Mosayebi-Samani M. (2020). Repeated stimulation of the dorsolateral-prefrontal cortex improves executive dysfunctions and craving in drug addiction: a randomized, double-blind, parallel-group study. *Brain Stimu.* 13 582–593. 10.1016/j.brs.2019.12.028 32289681

[B5] Allenby C. Falcone M. Bernardo L. Wileyto E. P. Rostain A. Ramsay J. R. (2018). Transcranial direct current brain stimulation decreases impulsivity in ADHD. *Brain Stimul.* 11 974–981. 10.1016/j.brs.2018.04.016 29885858 PMC6109423

[B6] Aron A. R. (2007). The neural basis of inhibition in cognitive control. *Neuroscientist* 13 214–228. 10.1177/1073858407299288 17519365

[B7] Aron A. R. Poldrack R. A. (2006). Cortical and subcortical contributions to stop signal response inhibition: role of the subthalamic nucleus. *J. Neurosci.* 26 2424–2433. 10.1523/JNEUROSCI.4682-05.2006 16510720 PMC6793670

[B8] Aron A. R. Robbins T. W. Poldrack R. A. (2014). Inhibition and the right inferior frontal cortex: one decade on. *Trends Cogn. Sci.* 18 177–185. 10.1016/j.tics.2013.12.003 24440116

[B9] Assecondi S. Hu R. Kroeker J. Eskes G. Shapiro K. (2022). Older adults with lower working memory capacity benefit from transcranial direct current stimulation when combined with working memory training: a preliminary study. *Front. Aging Neurosci.* 14:1009262. 10.3389/fnagi.2022.1009262 36299611 PMC9589058

[B10] Bari A. Robbins T. W. (2013). Inhibition and impulsivity: behavioral and neural basis of response control. *Prog. Neurobiol.* 108 44–79. 10.1016/j.pneurobio.2013.06.005 23856628

[B11] Berglund-Barraza A. Tian F. H. Basak C. Hart J. Evans J. L. (2020). Tracking changes in frontal lobe hemodynamic response in individual adults with developmental language disorder following HD tDCS enhanced phonological working memory training: an fNIRS feasibility study. *Front. Hum. Neurosci.* 14:15. 10.3389/fnhum.2020.00362 33132869 PMC7511756

[B12] Berkman E. Kahn L. Merchant J. (2014). Training-induced changes in inhibitory control network activity. *J. Neurosci.* 34 149–157. 10.1523/jneurosci.3564-13.2014 24381276 PMC3866481

[B13] Berryhill M. Martin D. (2018). Cognitive effects of transcranial direct current stimulation in healthy and clinical populations: an overview. *J. ECT* 34 e25–e35. 10.1097/yct.0000000000000534 30095685

[B14] Biggs A. T. Cain M. S. Mitroff S. R. (2015). Cognitive training can reduce civilian casualties in a simulated shooting environment. *Psychol. Sci.* 26 1164–1176. 10.1177/0956797615579274 26170262

[B15] Bikson M. Grossman P. Thomas C. Zannou A. Jiang J. Adnan T. (2016). Safety of transcranial direct current stimulation: evidence based update 2016. *Brain Stimul.* 9 641–661. 10.1016/j.brs.2016.06.004 27372845 PMC5007190

[B16] Boroda E. Krueger A. M. Bansal P. Schumacher M. J. Roy A. V. Boys C. J. (2020). A randomized controlled trial of transcranial direct-current stimulation and cognitive training in children with fetal alcohol spectrum disorder. *Brain Stimul.* 13 1059–1068. 10.1016/j.brs.2020.04.015 32360392

[B17] Breitling C. Zaehle T. Dannhauer M. Tegelbeckers J. Flechtner H.-H. Krauel K. (2020). Comparison between conventional and HD-tDCS of the right inferior frontal gyrus in children and adolescents with ADHD. *Clin. Neurophysiol.* 131 1146–1154. 10.1016/j.clinph.2019.12.412 32029377 PMC7250280

[B18] Brem A. K. Almquist J. N. F. Mansfield K. Plessow F. Sella F. Santarnecchi E. (2018). Modulating fluid intelligence performance through combined cognitive training and brain stimulation. *Neuropsychologia* 118 107–114. 10.1016/j.neuropsychologia.2018.04.008 29649503

[B19] Chiang H. S. Shakal S. Vanneste S. Kraut M. Hart J. (2021). Case report: improving verbal retrieval deficits with high definition transcranial direct current stimulation targeting the pre-supplementary motor area in a patient with chronic traumatic brain injury. *Front. Neurol.* 12:678518. 10.3389/fneur.2021.678518 34335447 PMC8322436

[B20] Cohen J. (1992). A power primer. *Psychol. Bull.* 112 155–159. 10.1037/0033-2909.112.1.155 19565683

[B21] Cohen Kadosh R. Soskic S. Iuculano T. Kanai R. Walsh V. (2010). Modulating neuronal activity produces specific and long-lasting changes in numerical competence. *Curr. Biol.* 20 2016–2020. 10.1016/j.cub.2010.10.007 21055945 PMC2990865

[B22] Congdon E. Mumford J. A. Cohen J. R. Adriana G. Turhan C. Poldrack R. A. (2012). Measurement and reliability of response inhibition. *Front. Psychol.* 3:37. 10.3389/fpsyg.2012.00037 22363308 PMC3283117

[B23] Corrêa, F. I. Carneiro Costa G. Leite Souza P. Marduy A. Parente J. Ferreira da Cruz S. (2022). Additive effect of transcranial direct current stimulation (tDCS) in combination with multicomponent training on elderly physical function capacity: a randomized, triple blind, controlled trial. *Physiother. Theory. Pract.* 1–14. 10.1080/09593985.2022.2081638 35619246

[B24] Dagan M. Herman T. Harrison R. Zhou J. Giladi N. Ruffini G. (2018). Multitarget transcranial direct current stimulation for freezing of gait in Parkinson’s disease. *Mov. Disord.* 33 642–646. 10.1002/mds.27300 29436740 PMC5964604

[B25] Dalley J. Robbins T. (2017). Fractionating impulsivity: neuropsychiatric implications. *Nat. Rev. Neurosci.* 18 158–171. 10.1038/nrn.2017.8 28209979

[B26] de Boer N. S. Schluter R. S. Daams J. G. van der Werf Y. D. Goudriaan A. E. van Holst R. J. (2021). The effect of non-invasive brain stimulation on executive functioning in healthy controls: a systematic review and meta-analysis. *Neurosci. Biobehav. Rev.* 125 122–147. 10.1016/j.neubiorev.2021.01.013 33503477

[B27] Di Rosa E. Brigadoi S. Cutini S. Tarantino V. Dell’Acqua R. Mapelli D. (2019). Reward motivation and neurostimulation interact to improve working memory performance in healthy older adults: a simultaneous tDCS-fNIRS study. *Neuroimage* 202:116062. 10.1016/j.neuroimage.2019.116062 31369810 PMC7467146

[B28] Diamond A. (2013). Executive functions. *Annu. Rev. Psychol.* 64 135–168. 10.1146/annurev-psych-113011-143750 23020641 PMC4084861

[B29] Ditye T. Jacobson L. Walsh V. Lavidor M. (2012). Modulating behavioral inhibition by tDCS combined with cognitive training. *Exp. Brain Res.* 219 363–368. 10.1007/s00221-012-3098-4 22532165

[B30] Dousset C. Ingels A. Schroder E. Angioletti L. Balconi M. Kornreich C. (2021). Transcranial direct current stimulation combined with cognitive training induces response inhibition facilitation through distinct neural responses according to the stimulation site: a follow-up event-related potentials study. *Clin. EEG Neurosci.* 52 181–192. 10.1177/1550059420958967 32924586

[B31] Duann J. R. Ide J. S. Luo X. Li C. S. (2009). Functional connectivity delineates distinct roles of the inferior frontal cortex and presupplementary motor area in stop signal inhibition. *J. Neurosci.* 29 10171–10179. 10.1523/JNEUROSCI.1300-09.2009 19675251 PMC2769086

[B32] Dubuson M. Kornreich C. Vanderhasselt M.-A. Baeken C. Wyckmans F. Dousset C. (2021). Transcranial direct current stimulation combined with alcohol cue inhibitory control training reduces the risk of early alcohol relapse: a randomized-controlled clinical trial. *Brain Stimul.* 14 1531–1543. 10.1016/j.brs.2021.10.386 34687964

[B33] Elmasry J. Loo C. Martin D. (2015). A systematic review of transcranial electrical stimulation combined with cognitive training. *Restorat. Neurol. Neurosci.* 33 263–278. 10.3233/rnn-140473 25624425

[B34] Fan J. McCandliss B. D. Sommer T. Raz A. Posner M. I. (2002). Testing the efficiency and independence of attentional networks. *J. Cogn. Neurosci.* 14 340–347. 10.1162/089892902317361886 11970796

[B35] Faul F. Erdfelder E. Lang A. Buchner A. (2007). G*Power 3: a flexible statistical power analysis program for the social, behavioral, and biomedical sciences. *Behavior. Res. Methods* 39 175–191. 10.3758/bf03193146 17695343

[B36] Filmer H. Varghese E. Hawkins G. Mattingley J. Dux P. (2017a). Improvements in attention and decision-making following combined behavioral training and brain stimulation. *Cereb. Cortex* 27 3675–3682. 10.1093/cercor/bhw189 27436130

[B37] Filmer H. L. Lyons M. Mattingley J. B. Dux P. E. (2017b). Anodal tDCS applied during multitasking training leads to transferable performance gains. *Sci. Rep.* 7:12988. 10.1038/s41598-017-13075-y 29021526 PMC5636876

[B38] Filmer H. L. Dux P. E. Mattingley J. B. (2014). Applications of transcranial direct current stimulation for understanding brain function. *Trends Neurosci.* 37 742–753. 10.1016/j.tins.2014.08.003 25189102

[B39] Forcano L. Mata F. de la Torre R. Verdejo-Garcia A. (2018). Cognitive and neuromodulation strategies for unhealthy eating and obesity: systematic review and discussion of neurocognitive mechanisms. *Neurosci. Biobehav. Rev.* 87 161–191. 10.1016/j.neubiorev.2018.02.003 29432784

[B40] Fu Z. Wu D. Ross I. Chung J. Mamelak A. Adolphs R. (2019). Single-neuron correlates of error monitoring and post-error adjustments in human medial frontal cortex. *Neuron* 101 165.e5–177.e5. 10.1016/j.neuron.2018.11.016 30528064 PMC6354767

[B41] Fujiyama H. Tan J. Puri R. Hinder M. R. (2021). Influence of tDCS over right inferior frontal gyrus and pre-supplementary motor area on perceptual decision-making and response inhibition: a healthy ageing perspective. *Neurobiol. Aging* 109 11–21. 10.1016/j.neurobiolaging.2021.09.014 34634749

[B42] Gbadeyan O. McMahon K. Steinhauser M. Meinzer M. (2016). Stimulation of dorsolateral prefrontal cortex enhances adaptive cognitive control: a high-definition transcranial direct current stimulation study. *J. Neurosci.* 36 12530–12536. 10.1523/jneurosci.2450-16.2016 27974612 PMC6705663

[B43] Gillespie S. M. Lee J. Williams R. Jones A. (2022). Psychopathy and response inhibition: a meta-analysis of go/no-go and stop signal task performance. *Neurosci. Biobehav. Rev.* 142 429–439. 10.1016/j.neubiorev.2022.104868 36113781

[B44] Gilmore C. S. Dickmann P. J. Nelson B. G. Lamberty G. J. Lim K. O. (2018). Transcranial direct current stimulation (tDCS) paired with a decision-making task reduces risk-taking in a clinically impulsive sample. *Brain Stimul.* 11 302–309. 10.1016/j.brs.2017.11.011 29174303

[B45] Goldin A. P. Hermida M. J. Shalom D. E. Elias Costa M. Lopez-Rosenfeld M. Segretin M. S. (2014). Far transfer to language and math of a short software-based gaming intervention. *Proc. Natl. Acad. Sci. U.S.A.* 111 6443–6448. 10.1073/pnas.1320217111 24711403 PMC4035955

[B46] Gregoret L. Zamorano A. M. Graven-Nielsen T. (2021). Effects of multifocal transcranial direct current stimulation targeting the motor network during prolonged experimental pain. *Eur. J. Pain* 25 1241–1253. 10.1002/ejp.1743 33539582

[B47] Guo Z. Gong Y. Lu H. Qiu R. Wang X. Zhu X. (2022a). Multitarget high-definition transcranial direct current stimulation improves response inhibition more than single-target high-definition transcranial direct current stimulation in healthy participants. *Front. Neurosci.* 16:905247. 10.3389/fnins.2022.905247 35968393 PMC9372262

[B48] Guo Z. Liang S. Ren L. Yang T. Qiu R. He Y. (2022b). Applying network analysis to understand the relationships between impulsivity and social media addiction and between impulsivity and problematic smartphone use. *Front. Psychiatry* 13:993328. 10.3389/fpsyt.2022.993328 36329911 PMC9623168

[B49] Han Y. M. Y. Chan M. M. Y. Shea C. K. S. Lai O. L. Krishnamurthy K. Cheung M. C. (2022). Neurophysiological and behavioral effects of multisession prefrontal tDCS and concurrent cognitive remediation training in patients with autism spectrum disorder (ASD): a double-blind, randomized controlled fNIRS study. *Brain Stimul.* 15 414–425. 10.1016/j.brs.2022.02.004 35181532

[B50] Hannah R. Aron A. R. (2021). Towards real-world generalizability of a circuit for action-stopping. *Nat. Rev. Neurosci.* 22 538–552. 10.1038/s41583-021-00485-1 34326532 PMC8972073

[B51] Hayes J. F. Osborn D. P. J. Lewis G. Dalman C. Lundin A. (2017). Association of late adolescent personality with risk for subsequent serious mental illness among men in a swedish nationwide cohort study. *JAMA Psychiatry* 74 703–711. 10.1001/jamapsychiatry.2017.0583 28538982 PMC5710245

[B52] Hill A. T. Rogasch N. C. Fitzgerald P. B. Hoy K. E. (2017). Effects of prefrontal bipolar and high-definition transcranial direct current stimulation on cortical reactivity and working memory in healthy adults. *Neuroimage* 152 142–157. 10.1016/j.neuroimage.2017.03.001 28274831

[B53] Hill A. T. Rogasch N. C. Fitzgerald P. B. Hoy K. E. (2018). Effects of single versus dual-site high-definition transcranial direct current stimulation (HD-tDCS) on cortical reactivity and working memory performance in healthy subjects. *Brain Stimul.* 11 1033–1043. 10.1016/j.brs.2018.06.005 29936014

[B54] Hogeveen J. Grafman J. Aboseria M. David A. Bikson M. Hauner K. K. (2016). Effects of high-definition and conventional tDCS on response inhibition. *Brain Stimul.* 9 720–729. 10.1016/j.brs.2016.04.015 27198577

[B55] Hsu T. Y. Tseng L. Y. Yu J. X. Kuo W. J. Hung D. L. Tzeng O. J. (2011). Modulating inhibitory control with direct current stimulation of the superior medial frontal cortex. *Neuroimage* 56 2249–2257. 10.1016/j.neuroimage.2011.03.059 21459149

[B56] Hughes M. Fulham W. Johnston P. Michie P. (2012). Stop-signal response inhibition in schizophrenia: behavioural, event-related potential and functional neuroimaging data. *Biol. Psychol.* 89 220–231. 10.1016/j.biopsycho.2011.10.013 22027085

[B57] Jacobson L. Javitt D. C. Lavidor M. (2011). Activation of inhibition: diminishing impulsive behavior by direct current stimulation over the inferior frontal gyrus. *J. Cogn. Neurosci.* 23 3380–3387. 10.1162/jocn_a_00020 21452949

[B58] Jurcak V. Tsuzuki D. Dan I. (2007). 10/20, 10/10, and 10/5 systems revisited: their validity as relative head-surface-based positioning systems. *Neuroimage* 34 1600–1611. 10.1016/j.neuroimage.2006.09.024 17207640

[B59] Kaminski J. Gleich T. Fukuda Y. Katthagen T. Gallinat J. Heinz A. (2020). Association of cortical glutamate and working memory activation in patients with schizophrenia: a multimodal proton magnetic resonance spectroscopy and functional magnetic resonance imaging study. *Biol. Psychiatry* 87 225–233. 10.1016/j.biopsych.2019.07.011 31521336

[B60] Kessler R. C. Adler L. Ames M. Demler O. Faraone S. Hiripi E. (2005). The World Health Organization adult ADHD self-report scale (ASRS): a short screening scale for use in the general population. *Psychol. Med.* 35 245–256. 10.1017/s0033291704002892 15841682

[B61] Kohl S. Hannah R. Rocchi L. Nord C. L. Rothwell J. Voon V. (2019). Cortical paired associative stimulation influences response inhibition: cortico-cortical and Cortico-subcortical networks. *Biol. Psychiatry* 85 355–363. 10.1016/j.biopsych.2018.03.009 29724490 PMC7004814

[B62] Krebs C. Peter J. Wyss P. Brem A. Klöppel S. (2021). Transcranial electrical stimulation improves cognitive training effects in healthy elderly adults with low cognitive performance. *Clin. Neurophysiol.* 132 1254–1263. 10.1016/j.clinph.2021.01.034 33875372

[B63] Kuo H.-I. Bikson M. Datta A. Minhas P. Paulus W. Kuo M.-F. (2013). Comparing cortical plasticity induced by conventional and high-definition 4 x 1 ring tDCS: a neurophysiological study. *Brain Stimul.* 6 644–648. 10.1016/j.brs.2012.09.010 23149292

[B64] Kwon Y. H. Kwon J. W. (2013a). Is transcranial direct current stimulation a potential method for improving response inhibition? *Neural Regenerat. Res.* 8 1048–1054. 10.3969/j.issn.1673-5374.2013.11.011 25206399 PMC4145879

[B65] Kwon Y. H. Kwon J. W. (2013b). Response inhibition induced in the stop-signal task by transcranial direct current stimulation of the pre-supplementary motor area and primary Sensoriomotor cortex. *J. Phys. Ther. Sci.* 25 1083–1086. 10.1589/jpts.25.1083 24259920 PMC3818760

[B66] Lee S.-A. Kim M.-K. (2021). The effect of transcranial direct current stimulation combined with visual cueing training on motor function, balance, and gait ability of patients with Parkinson’s disease. *Medicina* 57:1146. 10.3390/medicina57111146 34833364 PMC8617912

[B67] Li X. Y. Phillips M. R. Dong X. U. YaLi Z. ShaoJie Y. YongSheng T. (2011). Reliability and validity of an adapted Chinese version of Barratt Impulsiveness Scale. *Chinese Ment. Health J.* 25 610–615. 10.1007/s12583-011-0163-z

[B68] Lo K. Y. H. Hopman H. J. Chan S. C. Chau W. H. S. Cheng P. W. C. Cheung K. Y. (2022). Concurrent anodal transcranial direct current stimulation (tDCS) with cognitive training to improve cognition in schizophrenia. *Schizophr. Res.* 241 184–186. 10.1016/j.schres.2022.01.026 35131597

[B69] Logan G. D. Cowan W. B. Davis K. A. (1984). On the ability to inhibit simple and choice reaction time responses: a model and a method. *J. Exp. Psychol. Hum. Percept. Perform.* 10 276–291. 10.1037//0096-1523.10.2.276 6232345

[B70] Lu H. Gong Y. Huang P. Zhang Y. Guo Z. Zhu X. (2020a). Effect of repeated anodal HD-tDCS on executive functions: evidence from a pilot and single-blinded fNIRS study. *Front. Hum. Neurosci.* 14:583730. 10.3389/fnhum.2020.583730 33536886 PMC7847848

[B71] Lu H. Liu Q. Guo Z. Zhou G. Zhang Y. Zhu X. (2020b). Modulation of repeated anodal HD-tDCS on Attention in healthy young adults. *Front. Psychol.* 11:564447. 10.3389/fpsyg.2020.564447 33329194 PMC7714753

[B72] Martin D. M. Liu R. Alonzo A. Green M. Player M. J. Sachdev P. (2013). Can transcranial direct current stimulation enhance outcomes from cognitive training? A randomized controlled trial in healthy participants. *Int. J. Neuropsychopharmacol.* 16 1927–1936. 10.1017/s1461145713000539 23719048

[B73] McNab F. Leroux G. Strand F. Thorell L. Bergman S. Klingberg T. (2008). Common and unique components of inhibition and working memory: an fMRI, within-subjects investigation. *Neuropsychologia* 46 2668–2682. 10.1016/j.neuropsychologia.2008.04.023 18573510

[B74] Nejati V. Salehinejad M. A. Nitsche M. A. Najian A. Javadi A. H. (2020). Transcranial direct current stimulation improves executive dysfunctions in ADHD: implications for inhibitory control, interference control, working memory, and cognitive flexibility. *J. Attent. Disord.* 24 1928–1943. 10.1177/1087054717730611 28938852

[B75] Nitsche M. Paulus W. (2001). Sustained excitability elevations induced by transcranial DC motor cortex stimulation in humans. *Neurology* 57 1899–1901. 10.1212/wnl.57.10.1899 11723286

[B76] Nitsche M. A. Cohen L. G. Wassermann E. M. Priori A. Lang N. Antal A. (2008). Transcranial direct current stimulation: state of the art 2008. *Brain Stimul.* 1 206–223. 10.1016/j.brs.2008.06.004 20633386

[B77] Oldfield R. (1971). The assessment and analysis of handedness: the Edinburgh inventory. *Neuropsychologia* 9 97–113. 10.1016/0028-3932(71)90067-4 5146491

[B78] Owen A. M. McMillan K. M. Laird A. R. Bullmore E. (2005). N-back working memory paradigm: a meta-analysis of normative functional neuroimaging studies. *Hum. Brain Mapp.* 25 46–59. 10.1002/hbm.20131 15846822 PMC6871745

[B79] Paneri B. Adair D. Thomas C. Khadka N. Patel V. Tyler W. J. (2016). Tolerability of repeated application of transcranial electrical stimulation with limited outputs to healthy subjects. *Brain Stimul.* 9 740–754. 10.1016/j.brs.2016.05.008 27372844 PMC5786157

[B80] Parris B. Wadsley M. Arabaci G. Hasshim N. Augustinova M. Ferrand L. (2021). The effect of high-frequency rTMS of the left dorsolateral prefrontal cortex on the resolution of response, semantic and task conflict in the colour-word Stroop task. *Brain Struct. Funct.* 226 1241–1252. 10.1007/s00429-021-02237-4 33608822 PMC8036200

[B81] Patton J. H. Stanford M. S. Barratt E. S. (1995). Factor structure of the barratt impulsiveness scale. *J. Clin. Psychol.* 51 768–774. 8778124 10.1002/1097-4679(199511)51:6<768::aid-jclp2270510607>3.0.co;2-1

[B82] Pisano F. Manfredini A. Castellano A. Caltagirone C. Marangolo P. (2022). Does executive function training impact on communication? A randomized controlled tDCS study on post-stroke aphasia. *Brain Sci.* 12 1265. 10.3390/brainsci12091265 36139001 PMC9497246

[B83] Pisoni A. Mattavelli G. Papagno C. Rosanova M. Casali A. G. Romero Lauro L. J. (2018). Cognitive enhancement induced by anodal tDCS drives circuit-specific cortical plasticity. *Cereb. Cortex* 28 1132–1140. 10.1093/cercor/bhx021 28184424

[B84] Raiker J. S. Rapport M. D. Kofler M. J. Sarver D. E. (2012). Objectively-measured impulsivity and attention-deficit/hyperactivity disorder (ADHD): testing competing predictions from the working memory and behavioral inhibition models of ADHD. *J. Abnorm. Child Psychol.* 40 699–713. 10.1007/s10802-011-9607-2 22271141

[B85] Ran H. L. Fang D. Donald A. R. Wang R. Che Y. S. He X. T. (2021). Impulsivity mediates the association between parenting styles and self-harm in Chinese adolescents. *BMC Public Health* 21:332. 10.1186/s12889-021-10386-8 33568136 PMC7877034

[B86] Reinhart R. M. G. Nguyen J. A. (2019). Working memory revived in older adults by synchronizing rhythmic brain circuits. *Nat. Neurosci.* 22 820–827. 10.1038/s41593-019-0371-x 30962628 PMC6486414

[B87] Rinne P. Hassan M. Goniotakis D. Chohan K. Sharma P. Langdon D. (2013). Triple dissociation of attention networks in stroke according to lesion location. *Neurology* 81 812–820. 10.1212/WNL.0b013e3182a2ca34 23902704 PMC3908461

[B88] Sandrini M. Xu B. Volochayev R. Awosika O. Wang W. Butman J. (2020). Transcranial direct current stimulation facilitates response inhibition through dynamic modulation of the fronto-basal ganglia network. *Brain Stimul.* 13 96–104. 10.1016/j.brs.2019.08.004 31422052 PMC6889034

[B89] Schmicker M. Menze I. Schneider C. Taubert M. Zaehle T. Mueller N. G. (2021). Making the rich richer: frontoparietal tDCS enhances transfer effects of a single-session distractor inhibition training on working memory in high capacity individuals but reduces them in low capacity individuals. *NeuroImage* 242 118438–118438. 10.1016/j.neuroimage.2021.118438 34332042

[B90] Schneider N. Dagan M. Katz R. Thumm P. C. Brozgol M. Giladi N. (2021). Combining transcranial direct current stimulation with a motor-cognitive task: the impact on dual-task walking costs in older adults. *J. Neuroeng. Rehabil.* 18:23. 10.1186/s12984-021-00826-2 33526043 PMC7852224

[B91] Sehatpour P. Dondé C. Adair D. Kreither J. Lopez-Calderon J. Avissar M. (2021). Comparison of cortical network effects of high-definition and conventional tDCS during visuomotor processing. *Brain Stimul.* 14 33–35. 10.1016/j.brs.2020.11.004 33181350

[B92] Sharma M. Farahani F. Bikson M. Parra L. C. (2021). Weak DCS causes a relatively strong cumulative boost of synaptic plasticity with spaced learning. *Brain Stimul.* 15 57–62. 10.1016/j.brs.2021.10.552 34749007 PMC8816825

[B93] Shaw J. S. Hosseini S. M. H. (2021). The effect of baseline performance and age on cognitive training improvements in older adults: a qualitative review. *J. Prev. Alzheimers Dis.* 8 100–109. 10.14283/jpad.2020.55 33336231 PMC8290874

[B94] Shen B. Yin Y. Wang J. Zhou X. McClure S. M. Li J. (2016). High-definition tDCS alters impulsivity in a baseline-dependent manner. *Neuroimage* 143 343–352. 10.1016/j.neuroimage.2016.09.006 27608604

[B95] Smits F. M. Geuze E. Schutter D. van Honk J. Gladwin T. E. (2021). Effects of tDCS during inhibitory control training on performance and PTSD, aggression and anxiety symptoms: a randomized-controlled trial in a military sample. *Psychol. Med.* 52 1–11. 10.1017/s0033291721000817 33757606 PMC9811348

[B96] Song S. Zilverstand A. Gui W. Li H. J. Zhou X. (2019). Effects of single-session versus multi-session non-invasive brain stimulation on craving and consumption in individuals with drug addiction, eating disorders or obesity: a meta-analysis. *Brain Stimul.* 12 606–618. 10.1016/j.brs.2018.12.975 30612944

[B97] Stephens J. A. Berryhill M. E. (2016). Older adults improve on everyday tasks after working memory training and neurostimulation. *Brain Stimul.* 9 553–559. 10.1016/j.brs.2016.04.001 27178247 PMC4957521

[B98] Stramaccia D. F. Penolazzi B. Sartori G. Braga M. Mondini S. Galfano G. (2015). Assessing the effects of tDCS over a delayed response inhibition task by targeting the right inferior frontal gyrus and right dorsolateral prefrontal cortex. *Exp. Brain Res.* 233 2283–2290. 10.1007/s00221-015-4297-6 25925996

[B99] Stroop J. R. (1935). Studies on interference in serial verbal reactions. *J. Exp. Psychol.* 18 643–662.

[B100] Szymkowicz S. M. Taylor W. D. Woods A. J. (2022). Augmenting cognitive training with bifrontal tDCS decreases subclinical depressive symptoms in older adults: preliminary findings. *Brain Stimul.* 15 1037–1039. 10.1016/j.brs.2022.07.055 35931378 PMC9637028

[B101] Teixeira-Santos A. C. Moreira C. S. Pereira D. R. Pinal D. Fregni F. Leite J. (2022). Working memory training coupled with transcranial direct current stimulation in older adults: a randomized controlled experiment. *Front. Aging Neurosci.* 14:827188. 10.3389/fnagi.2022.827188 35493937 PMC9039392

[B102] Turski C. A. Kessler-Jones A. Chow C. Hermann B. Hsu D. Jones J. (2017). Extended multiple-field high-definition transcranial direct current stimulation (HD-tDCS) is well tolerated and safe in healthy adults. *Restorat. Neurol. Neurosci.* 35 631–642. 10.3233/rnn-170757 29172010 PMC5730273

[B103] Val-Laillet D. Aarts E. Weber B. Ferrari M. Quaresima V. Stoeckel L. E. (2015). Neuroimaging and neuromodulation approaches to study eating behavior and prevent and treat eating disorders and obesity. *Neuroimage Clin.* 8 1–31. 10.1016/j.nicl.2015.03.016 26110109 PMC4473270

[B104] van Rooij D. Hoekstra P. J. Mennes M. von Rhein D. Thissen A. J. Heslenfeld D. (2015). Distinguishing adolescents With ADHD from their unaffected siblings and healthy comparison subjects by neural activation patterns during response inhibition. *Am. J. Psychiatry* 172 674–683. 10.1176/appi.ajp.2014.13121635 25615565 PMC4490085

[B105] Verbruggen F. Aron A. R. Band G. P. H. Beste C. Bissett P. G. Brockett A. T. (2019). A consensus guide to capturing the ability to inhibit actions and impulsive behaviors in the stop-signal task. *eLife* 8:e46323. 10.7554/eLife.46323 31033438 PMC6533084

[B106] Verbruggen F. Logan G. D. (2008). Response inhibition in the stop-signal paradigm. *Trends Cogn. Sci.* 12 418–424. 10.1016/j.tics.2008.07.005 18799345 PMC2709177

[B107] Verbruggen F. Logan G. D. (2009). Models of response inhibition in the stop-signal and stop-change paradigms. *Neurosci. Biobehav. Rev.* 33 647–661. 10.1016/j.neubiorev.2008.08.014 18822313 PMC2696813

[B108] Villamar M. F. Volz M. S. Bikson M. Datta A. DaSilva A. F. Fregni F. (2013). Technique and considerations in the use of 4x1 ring high-definition transcranial direct current stimulation (HD-tDCS). *J. Vis. Exp.* e50309. 10.3791/50309 23893039 PMC3735368

[B109] Watanabe T. Hanajima R. Shirota Y. Tsutsumi R. Shimizu T. Hayashi T. (2015). Effects of rTMS of pre-supplementary motor area on fronto basal ganglia network activity during stop-signal task. *J. Neurosci.* 35 4813–4823. 10.1523/jneurosci.3761-14.2015 25810512 PMC6705371

[B110] Weidler C. Habel U. Wallheinke P. Wagels L. Hofhansel L. Ling S. C. (2022). Consequences of prefrontal tDCS on inhibitory control and reactive aggression. *Soc. Cogn. Affect. Neurosci.* 17 120–130. 10.1093/scan/nsaa158 33227131 PMC8824612

[B111] Westwood S. J. Criaud M. Lam S. L. Lukito S. Wallace-Hanlon S. Kowalczyk O. S. (2021). Transcranial direct current stimulation (tDCS) combined with cognitive training in adolescent boys with ADHD: a double-blind, randomised, sham-controlled trial. *Psychol. Med.* 53 1–16. 10.1017/s0033291721001859 34225830 PMC9899574

[B112] Whelan R. Conrod P. Poline J. Lourdusamy A. Banaschewski T. Barker G. (2012). Adolescent impulsivity phenotypes characterized by distinct brain networks. *Nat. Neurosci.* 15 920–925. 10.1038/nn.3092 22544311

[B113] Wilkinson S. T. Holtzheimer P. E. Gao S. Kirwin D. S. Price R. B. (2019). Leveraging neuroplasticity to enhance adaptive learning: the potential for synergistic somatic-behavioral treatment combinations to improve clinical outcomes in depression. *Biol. Psychiatry* 85 454–465. 10.1016/j.biopsych.2018.09.004 30528745 PMC6380941

[B114] Wu D. Zhou Y. Xu P. Liu N. Sun K. Xiao W. (2021b). Initial performance modulates the effects of cathodal transcranial direct current stimulation (tDCS) over the right dorsolateral prefrontal cortex on inhibitory control. *Brain Res.* 1774:147722. 10.1016/j.brainres.2021.147722 34774867

[B115] Wu D. Zhang P. Liu N. Sun K. Xiao W. (2021a). Effects of high-definition transcranial direct current stimulation over the left fusiform face area on face view discrimination depend on the individual baseline performance. *Front. Neurosci.* 15:704880. 10.3389/fnins.2021.704880 34867146 PMC8639859

[B116] Xu P. Wu D. Chen Y. Wang Z. Xiao W. (2020). The effect of response inhibition training on risky decision-making task performance. *Front. Psychol.* 11:1806. 10.3389/fpsyg.2020.01806 32793080 PMC7393991

[B117] Yavari F. Jamil A. Samani M. M. Vidor L. P. Nitsche M. A. (2018). Basic and functional effects of transcranial electrical stimulation (tES)-an introduction. *Neurosci. Biobehav. Rev.* 85 81–92. 10.1016/j.neubiorev.2017.06.015 28688701

[B118] Yeh C. B. Gau S. S. Kessler R. C. Wu Y. Y. (2008). Psychometric properties of the Chinese version of the adult ADHD self-report scale. *Int. J. Methods Psychiatr. Res.* 17 45–54. 10.1002/mpr.241 18286465 PMC6878254

[B119] Zhao X. Chen L. Maes J. (2018). Training and transfer effects of response inhibition training in children and adults. *Dev. Sci.* 21. 10.1111/desc.12511 27966279

[B120] Zhou J. Manor B. Yu W. Lo O. Y. Gouskova N. Salvador R. (2021). Targeted tDCS mitigates dual-task costs to gait and balance in older adults. *Ann. Neurol.* 90 428–439. 10.1002/ana.26156 34216034 PMC8434977

[B121] Zhou J. Xuan B. (2022). Inhibitory control training and transcranial direct current stimulation of the pre-supplementary motor area: behavioral gains and transfer effects. *Exp. Brain Res.* 240 909–925. 10.1007/s00221-021-06297-0 35083548

