# Supplementary material for: Long-term effects of repeated multitarget high-definition transcranial direct current stimulation combined with cognitive training on response inhibition gains
Source: Front Neurosci. 2023 Mar 9;17:1107116. doi: 10.3389/fnins.2023.1107116 (PMC10033537; doi:10.3389/fnins.2023.1107116)
Supplement: Supplementary file 1 [file Data_Sheet_1.docx]

Supplementary Material


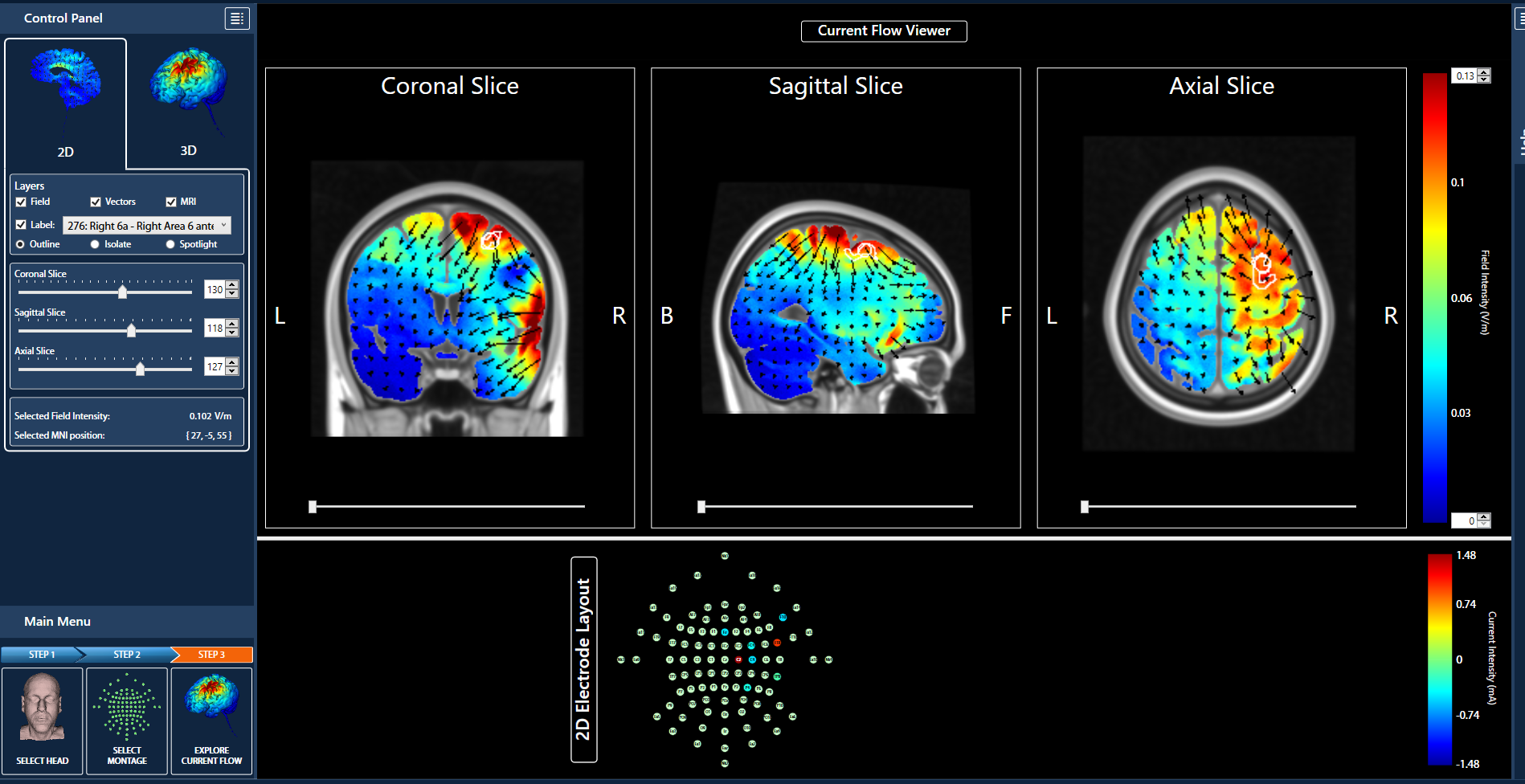


Figure 1 Area 6a


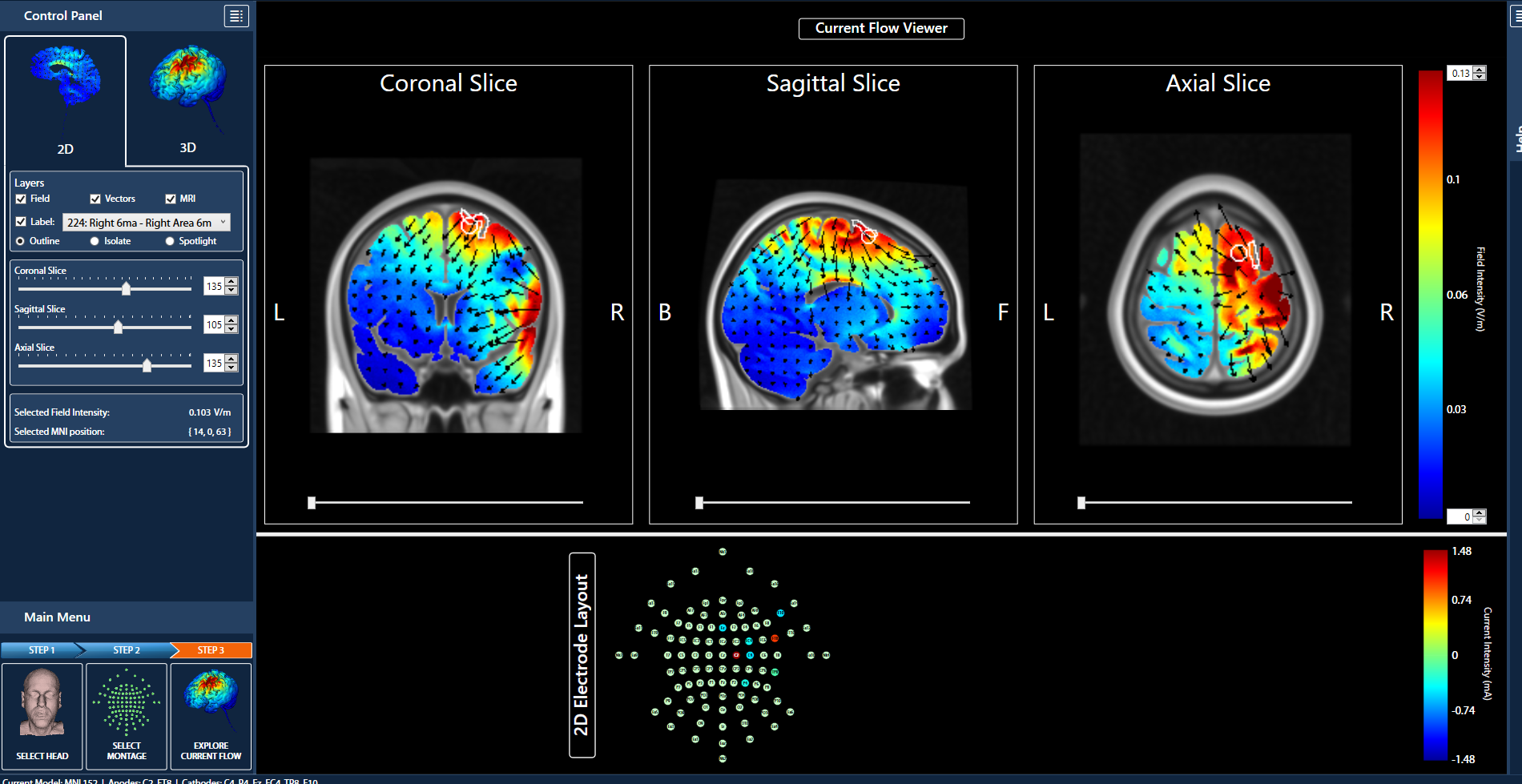


Figure 2 Area 6ma


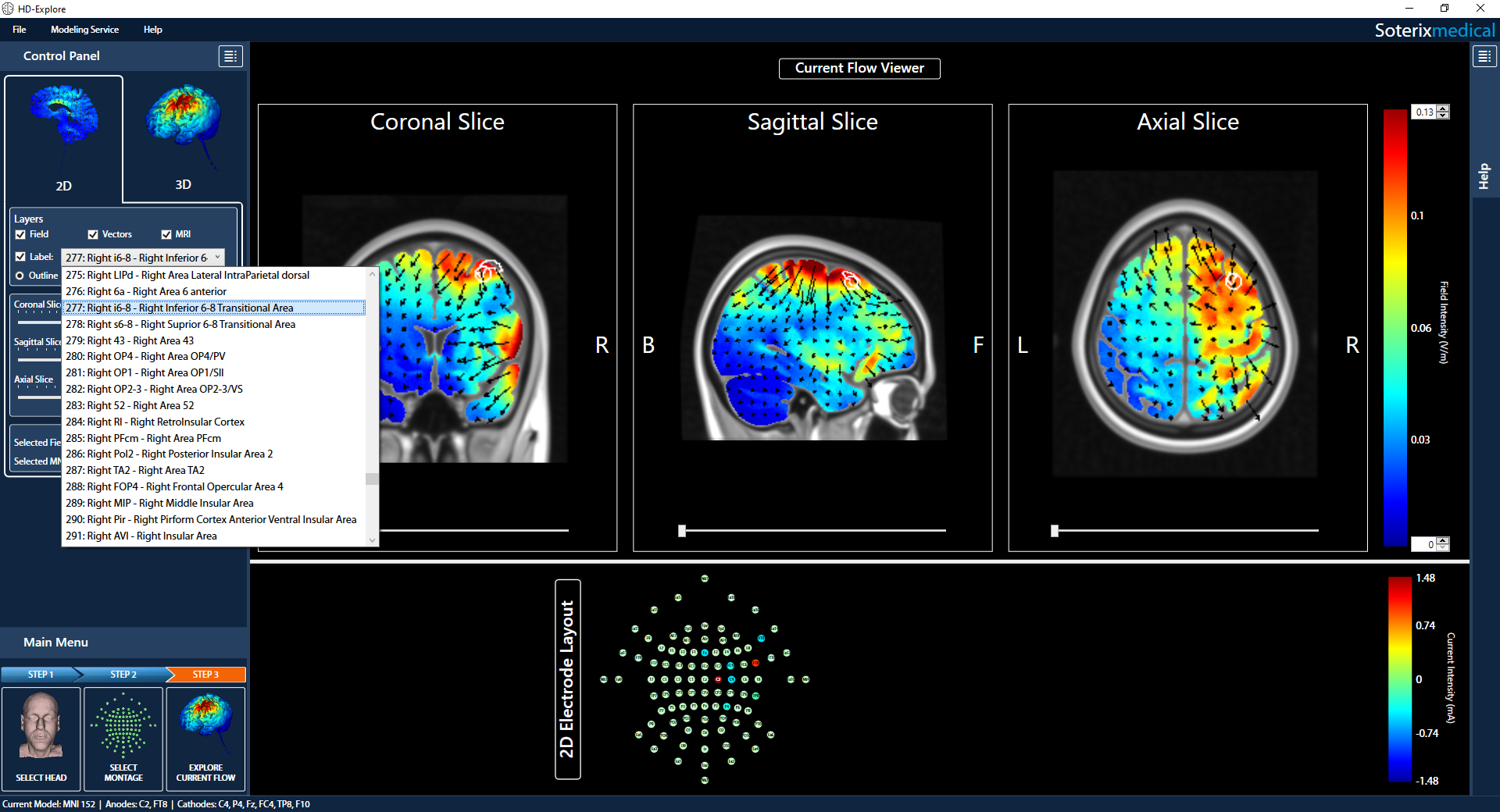


Figure 3 Area i6-8


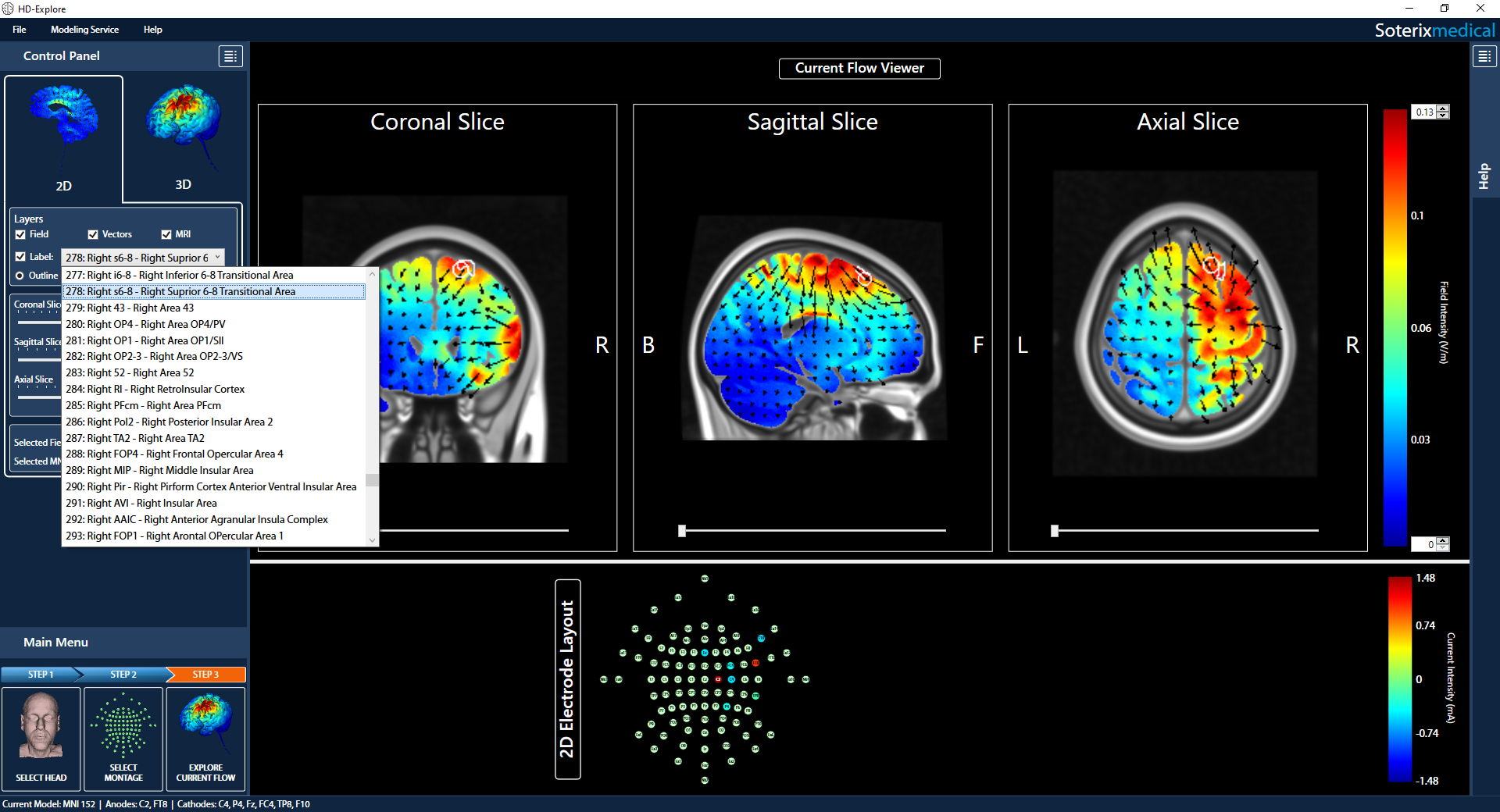


Figure 4 Area s6-8


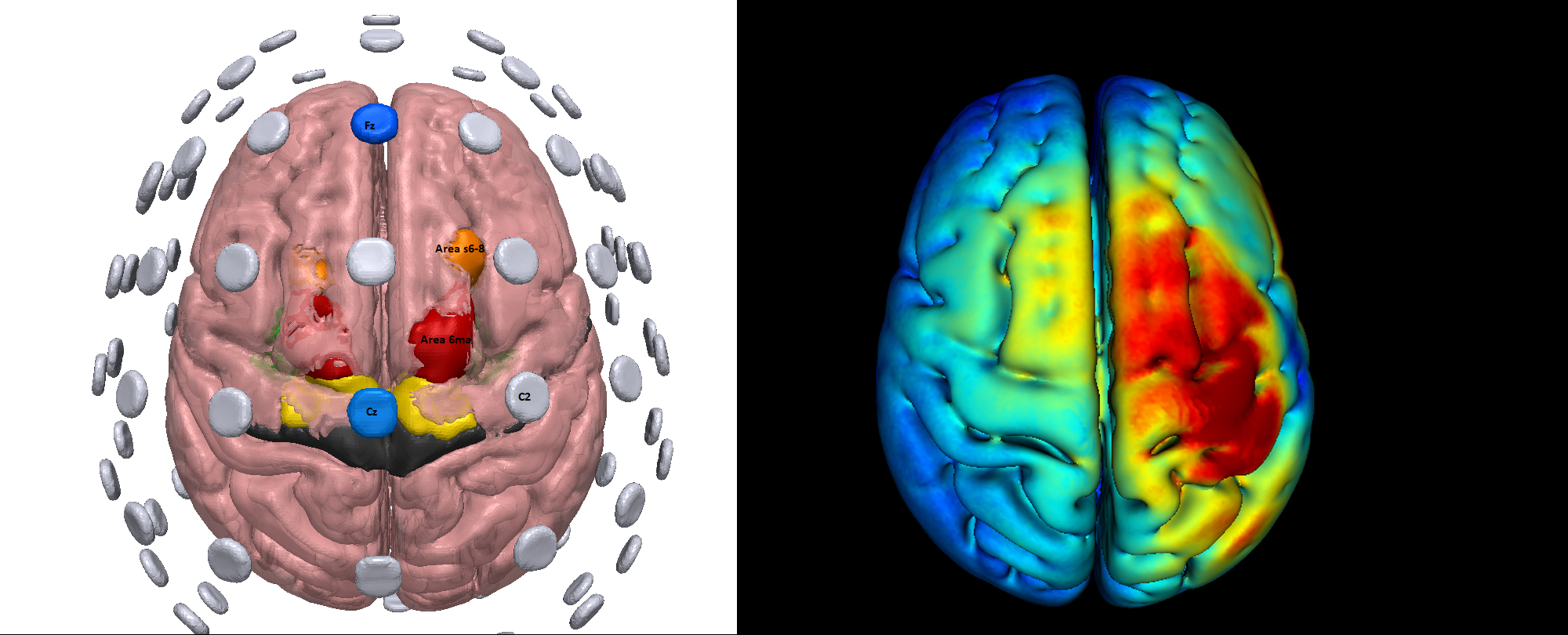


Figure 5 Electrode map and top view 3D modeling
